# Supplementary material for: Hypotension after general anesthesia induction using remimazolam in geriatric patients: Protocol for a double-blind randomized controlled trial
Source: PLoS One. 2022 Sep 30;17(9):e0275451. doi: 10.1371/journal.pone.0275451 (PMC9524631; doi:10.1371/journal.pone.0275451)
Supplement: S2 File — (DOCX) [file pone.0275451.s002.docx]

**Supporting information 2. Definition and evaluation of adverse events**

**Definition of adverse events (AEs)**

AEs are defined as all unfavorable or unintended medical events that occur in research patients, whether or not causally related to the research protocol. In this study, events that occurred between the start of induction of general anesthesia and the end of the follow-up period will be treated as AEs, and data on serious AEs and Grade 2 or higher AEs will be recorded. All AEs must be followed up until resolving or stabilization at an acceptable level by the investigator. The investigators should describe about the adverse event name, date of occurrence, severity, treatments, and outcome (*i.e.*, describe the time of recovery, or the time when symptoms are fixed) to the medical records. If the investigators will become aware of the occurrence of AEs, he or she will report it to the principal investigator. The principal investigator reports the AEs to the responsible person of the research institution, as necessary, in accordance with the procedure manual of the research institution.

**Evaluation of AEs**

The degree of AEs is evaluated according to Common Terminology Criteria for Adverse Event v4.0.

**Grade 1**, Mild (no need for intervention for AEs)

**Grade 2**, Moderate (AEs requires intervention such as outpatient drug treatment)

**Grade 3**, Severe (AEs requires intervention such as inpatient treatment)

**Grade 4**, Life-threatening or incapacitated

**Grade 5**, Death

The relationship to the study drug is evaluated as follows.

**Related:** The causal relationship with protocol of this study is valid and may not be due to exacerbations of primary disease, complications, or other treatments.

**Suspected:** The causal relationship with protocol is unclear. It can also be explained by exacerbations of the primary disease, complications, and other treatments.

**Not relevant:** It is not causally related to the protocol and can be clearly explained by exacerbations of the primary disease, complications, other treatments.

**Definition of serious AEs**

1. Results in death
2. Life threatening
3. Requires or prolongs patient hospitalization
4. Permanent or significant disability or incapacity
5. Congenital anomaly or birth defect
6. Other medically important conditions (*i.e.*, requires any intervention to prevent one of the previously listed outcomes)
