# Supplementary material for: Hypotension after general anesthesia induction using remimazolam in geriatric patients: Protocol for a double-blind randomized controlled trial
Source: PLoS One. 2022 Sep 30;17(9):e0275451. doi: 10.1371/journal.pone.0275451 (PMC9524631; doi:10.1371/journal.pone.0275451)
Supplement: S3 File — (DOCX) [file pone.0275451.s003.docx]

**Research Protocol**

**Comparison of the incidence of hypotension during general anesthesia induction between remimazolam and propofol: A protocol for a double-blind randomized controlled trial**

**1. Object and meaning**

In patients aged ≥80 years who will undergo elective surgery under general anesthesia, the incidence of hypotension during induction using remimazolam will be compared in a group using propofol as a control.

**2. Evidence of scientific rationality of research**

**2.1. Explanation of the target disease**

In Japan, the proportion of elderly people is increasing. Therefore, the number of surgeries for elderly patients will also increase. Because elderly patients tend to have a decline in physical and physiological functions and have more comorbidities, anesthetists must be managed more carefully than younger patients. According to a survey on the investigation of anesthesia-related complications in 2018 by the Japanese Society of Anesthesiologists, the frequency of cardiac arrest and severe hypotension began to increase in the in patients aged >80 years and was highest in patients aged >90 years except in the neonatal period <1 month. The frequency of hypotension during anesthesia is higher in the elderly than in other age groups, and increasing age is associated with a higher frequency of hypotension during the first 20 min after induction of general anesthesia. Hypotension during general anesthesia has been suggested to be associated with poor outcomes, such as organ dysfunction or perioperative mortality. Therefore, it is clinically meaningful to verify the occurrence of hypotension with remimazolam compared with controls, especially in the elderly. A reduction in the occurrence of hypotension is expected to provide better quality care to the elderly and reduce medical costs.

**2.2. Control treatment**

Propofol, which has a rapid onset and a few side effects, is one of the standard sedatives for induction of general anesthesia. However, propofol has some disadvantages, such as vasodilation or reduction in cardiac output. Age-related physiological and physical changes, such as autonomic nervous system dysfunction and arteriosclerosis, tend to cause propofol-induced hypotension, especially in older patients compared with other age groups. The package insert for propofol states that it should be used with caution in patients with American Society of Anesthesiologists (ASA) risk category III/IV and elderly patients and recommends slowing down the rate of administration to 1/2 of the usual dose, or 0.25 mg/kg/10 s.

**2.3. Trial treatment**

Remimazolam, approved for the induction and maintenance of general anesthesia or procedural sedation, is a full agonist at the benzodiazepine-binding site of the GABA A receptor. Remimazolam is a novel, short-acting drug because it is rapidly metabolized by nonspecific tissue esterases, and its metabolites have no drug activity. Remimazolam was noninferior to propofol in terms of efficacy as a hypnotic for general anesthesia. The incidence of hypotension that required treatment during general anesthesia in the remimazolam group tended to be lower than in the propofol group (33% vs. 60%). In a trial investigating the efficacy and safety of remimazolam collected in ASA PS of class III or higher, the incidence of decreased blood pressure throughout the trial period was 67.7% when remimazolam was administered at 12 mg/kg/h for induction. However, to our knowledge, no randomized controlled trials have examined the incidence of hypotension during general anesthesia induction using remimazolam in elderly patients as the primary outcome.

**2.4. Trial design and primary endpoints**

This is a single-center, double-blind, randomized, two-arm parallel-group, standard treatment-controlled, interventional clinical study.

Many factors are associated with the hemodynamic fluctuations caused by general anesthesia. We plan our study design as a prospective randomized control trial because the number of cases required to adjust all these factors based on observational studies is enormous. Due to the potential for arbitrary dropouts and preconceived evaluation of outcomes, a double-blind study design was adopted. Blinding of the patient is done to prevent the anesthetists and evaluator from breaking the blinding by unexpectedly vocalizing the drug name.

**Primary outcome:** The primary outcome will be the incidence of hypotension between the start of propofol or remimazolam and 3 min after tracheal intubation. Hypotension was defined as a mean arterial pressure <65 mmHg, as measured in the brachial artery using a manchette. Mean arterial pressure was selected as the primary outcome because a mean arterial pressure <65 mmHg sustained for more than 5 min during general anesthesia has been suggested to be associated with the occurrence of perioperative organ damage, such as cardiac and renal, and perioperative mortality.

**2.5. Significance of this study**

The proportion of elderly people in Japan is increasing. The demand for surgery for elderly patients is also increasing. Elderly patients tend to have a decline in physical and physiological functions and tend to have more comorbidities. Due to the high risk of perioperative complications, anesthesiologists should manage general anesthesia in elderly patients more carefully than in younger patients. Age-related physiological and physical changes tend to cause propofol-induced hypotension compared to other age groups, especially in older patients. Therefore, it is clinically meaningful to verify the occurrence of hypotension with remimazolam compared with controls, especially in the elderly. A reduction in the occurrence of hypotension is expected to provide better quality care to the elderly and reduce medical costs. If the results are negative, it indicates that there is little need to change the current clinical practice.

**3. Drug information**

**3.1. Trial drug**

**Product name:** Anerem

**Generic name:** Remimazolam besilate

**Manufacturing and sales company:** Mundipharma K.K.

**Indications:** Induction and management of general anesthesia

**Dosage and administration:** The dose for adult remimazolam is 12 mg/kg/hour by continuous intravenous infusion until loss of consciousness while monitoring the patient's condition.

**Drug form:** Injection drug (vial)

**3.2. Control drug**

**Generic name:** Propofol

**Indications:** Induction and management of general anesthesia

**Dosage and administration:** In adults, the drug should be administered intravenously at a rate of 0.05 mL/kg/10 s (0.5 mg/kg/10 s as propofol) while monitoring the patient's condition until loss of consciousness. In patients with ASA III or IV, it should be administered slowly. In adults, 0.20−0.25 mL/kg (2.0−2.5 mg/kg of propofol) is sufficient to achieve loss of consciousness. In the elderly, a smaller dose may be sufficient to achieve loss of consciousness.

**Dosage and administration for the elderly:** Because elderly patients often have liver, renal, or baroreceptor reflex dysfunction, side effects concerning cardiovascular are more likely to occur. This drug should be administered cautiously while observing the patient's condition (*e.g.*, slowing the administration rate at induction to 1/2, *i.e.*, approximately 0.025 mL/kg/10 s).

**Drug form:** Injection drug (vial or ample)

**4. Criteria and definitions used in this study**

Not applicable.

**5. Selection criteria for research participants**

**5.1. Inclusion criteria**

1. Patients aged ≥80 years who will undergo elective noncardiovascular surgery under general anesthesia
2. Patients who will be performed using rapid induction of general anesthesia with oral intubation
3. Patients who can communicate and obtain consent from the patient
4. Patients with an ASA physical status rating of class Ⅰ to Ⅲ

**5.2. Exclusion criteria**

1. Patients with a cerebral aneurysm or thoracic or abdominal aortic aneurysm
2. Patients with predicted or history of difficulty in airway management
3. Patients with drug allergies associated with this study
4. Patients on maintenance dialysis
5. Patients with severe liver dysfunction (*i.e.*, Child−Pugh class C)
6. Untreated and unstable ischemic heart disease
7. Severe aortic/mitral valvular disease
8. Arterial fibrillation or multiple premature atrial contractions or premature ventricular contractions
9. Congestive heart failure (*i.e.*, New York Heart Association classification Ⅲ or Ⅳ)
10. Pregnant women
11. Body mass index ≥30
12. Patients who regularly use antipsychotics or antidepressant drugs
13. Patients judged inappropriate for participation by researchers
14. Patients with mean blood pressure before induction of general anesthesia less than 70 mmHg*
15. Patients who failed the first attempt of tracheal intubation*

*Even after enrollment, it will not be included in the dropout or discontinuation but will be treated as an excluded case.

**6. Research plan**

**6.1. Design**

This is a single-center, double-blind, randomized, two-arm parallel-group, standard treatment-controlled, interventional clinical study.

**6.2. Sample size**

Ninety cases (standard treatment group: 45 cases and trial drug group: 45 cases)

**6.3. Study period**

**Study period:** From the date of approval for implementation until March 31, 2025

**Registration period:** From the date of approval for implementation until September 30, 2022

**Follow-up period:** Up to the day after surgery for each case

**6.4.** **Facility registration and case registration/allocation method**

**6.4.1. Registration center**

**Person in charge:** Yusuke Mizuno

**Facility Name:** Department of Anesthesiology, Yokohama City University Hospital

**Office hours:** 8:30−17:15 on weekdays (not available on Saturdays, Sundays, national holidays, and year-end and New Year holidays).

**6.4.2. Facility registration**

Not applicable (single-center study).

**6.4.3. Enrollment and allocation**

- 1. Blinded researchers will screen potentially eligible participants from the list of operations up to 1-day before surgery. Participants will be checked for inclusion and exclusion criteria.
  2. The principal investigator or subinvestigators fill out the case registration form. All participants will be identified by individual registry numbers used to anonymize the care report form or other documents. This individual registry number consists of numbers or letters not related to information that can identify a specific individual.
  3. Submit the case registration form to the registration center. The registration center checks the case registration form, and if eligibility is reconfirmed, proceeds to allocation.
  4. The **unblinded physician** determined the trial drug from the allocation sequence list. An **unblinded physician** is defined as one who is not consistently involved in patient recruitment, data collection, or anesthesia management during the study period, but only in drug administration.
  5. The research office will archive the registration confirmation form.

**6.4.4. Allocation and stratification factor**

The allocation will be performed by an unblinded researcher before the preparation of anesthesia. The unblinded researcher prepares the allocation list before the start of the first case registration and keeps it in a locked cabinet. The allocation sequence list is generated using computer-generated random numbers. The key will be managed so that anesthetists, data collectors, and data analysts will not be able to access it until the analysis is completed. The results of the allocation will not be made known to anesthetists or evaluators.

Our study will use permuted block randomization with stratification based on the absence or presence of hypertension, defined as systolic blood pressure (SBP) measured at a ward ≥140 mmHg or diastolic blood pressure ≥90 mmHg. The detailed procedure for random allocation will not be communicated to blinded investigators.

**The basis for stratification factor:** Participants with a lower usual blood pressure may meet the mean blood pressure <65 mmHg after induction of general anesthesia more often than those without.

**6.4.5. Blinding**

In this study, unblinded researchers were responsible for the preparation and administration of the trial drugs. Trial drugs (*i.e.*, propofol or remimazolam) will be prepared before starting anesthesia on the day of surgery. This unblinded researcher will be dedicated to allocation, drug preparation, and administration and will not participate in other tasks such as recruitment, data collection, an anesthetist in charge, or an evaluator.

The syringe pump with trial drugs, covered with an opaque plastic bag, will be brought to the operating room by an unblinded researcher. After the participant will be laid on the bed, the syringe pump with a trial drug, IV line, and puncture site of the IV line of the participant’s forearm will be blinded to other researchers and the participant using towels, a partition, and opaque plastic sheets. After these procedures, another blinded researcher will enter the operating room and then will start the induction of general anesthesia.

**6.4.6. Unblinding procedures**

If serious adverse events (SAEs) such as a medical emergency, which requires identifying an intervention of individual participants, blinded investigators are permitted to inquire into unblinded investigators.

**6.5. Treatment plan**

**6.5.1. Protocol treatment**

**6.5.1.1. Trial drug group**

Remimazolam (12 mg/kg/h) will be continuously infused using a syringe pump until loss of consciousness, and then sevoflurane 1.5% will be started for maintenance of anesthesia. Three minutes after loss of consciousness, tracheal intubation was performed using a McGRATH MAC video laryngoscope.

**6.5.1.2. Control group**

Propofol (0.25 mg/kg/10 s) will be continuously infused using a syringe pump until loss of consciousness, and then sevoflurane 1.5% will be started as maintenance of anesthesia. Three minutes after loss of consciousness, tracheal intubation will be performed using a McGRATH MAC video laryngoscope.

**6.5.2. Concomitant medication and therapy**

**6.5.2.1. Concomitant medication and therapy**

**Remifentanil:** It will start at 0.25 mcg/kg/min before administering the trial or control drug. After tracheal intubation, remifentanil will be immediately decelerated to 0.05 mcg/kg/h.

**Rocuronium:** Rocuronium (0.6 mg/kg) will be administered after confirming loss of consciousness.

**Sevoflurane:** After confirming the loss of consciousness, sevoflurane 1.5% will be administered as a maintenance dose.

**6.5.2.2. No concomitant treatment**

Sedatives used for the induction of general anesthesia other than protocol treatment

**6.5.2.3. Treatments that can be used in combination**

**Hypotension**

a) Hypotension with heart rate <80 beats/min, ephedrine 4 mg

b) Hypotension with heart rate ≥80 beats/min, phenylephrine 0.05 mg.

**Bradycardia** (heart rate <45 beats/min)

a) Bradycardia without severe hypotension, atropine 0.5 mg bolus (one additional dose allowed.)

b) Bradycardia with hypotension and ephedrine 4 mg is acceptable in addition to atropine.

**Others**

Drugs for the treatment of complications and adverse events (AEs)

**6.5.3. Criteria for dose reduction and drug withdrawal**

Discontinue the treatment protocol if the physician in charge determines that it is difficult to continue treatment due to the occurrence of AEs.

**6.5.4. Criteria for dose increase and resumption**

Not applicable

**6.5.5. Discontinuation of research in individual research participants**

**6.5.5.1. Discontinuation criteria**

- 1. Participant's request to withdraw consent
  2. Cases that are determined after enrollment to be inappropriate for inclusion because they do not meet the inclusion criteria or conflict with the exclusion criteria
  3. Severe hemodynamic instability (*i.e.*, hypotension with poor response to vasopressor use, severe bradycardia) between anesthesia induction and observation period
  4. In case of difficulty in continuation due to the occurrence of AEs
  5. Serious deviations from the protocol
  6. The decision by investigators and others

**6.5.5.2. Discontinuation procedure**

- 1. If the administration of the trial drug is to be discontinued, the necessary observations, examinations, and evaluation will be made. Participants will be informed after awakening from anesthesia.
  2. Appropriate treatments will be taken for the participant when the administration of the trial drug is discontinued due to the occurrence of AEs or other safety issues. Continue follow-up and document outcomes until symptoms (including laboratory values) return to pretrial drug or baseline status or until symptoms stabilize.
  3. The reason for the discontinuation of administration should be stated in the case report form (CRF).

**6.5.5.3. Posttreatment after discontinuation of protocol treatment**

Not applicable

**7. Observation, examination, investigation, and evaluation items**

**7.1. Participant’s timeline**

|  | **STUDY PERIOD** | | | | | | |
| --- | --- | --- | --- | --- | --- | --- | --- |
|  | **Enrollment** | **Allocation** | **Post-allocation** | | | | **Close-out** |
| **TIMEPOINT*** | **1** | **2** | **3** | **4** | **5** | **6** | **7** |
| **ENROLLMENT:**  **Eligibility screen** | **X** |  |  |  |  |  |  |
| **Informed consent** | **X** |  |  |  |  |  |  |
| **Allocation** |  | **X** |  |  |  |  |  |
| **INTERVENTIONS: *[Remimazolam]*** |  |  |  | **X** |  |  |  |
| ***[Propofol]*** |  |  |  | **X** |  |  |  |
| **ASSESSMENTS:** |  |  |  |  |  |  |  |
| ***Baseline variables***  ***[age, sex, height, weight, medical history, medicines, ASA PS, CFS, surgical procedure, laboratory values]*** | **X** | **X** |  |  |  |  |  |
| ***Vital sings***  ***[blood pressure, heart rate]*** | **X** |  | **X** | **X** | **X** | **X** |  |
| ***Time to loss of consciousness*** |  |  |  | **X** |  |  |  |
| ***Induction drug injection pain*** |  |  |  | **X** |  |  |  |
| ***Dose of trial drugs for induction*** |  |  |  |  |  | **X** |  |
| ***Dose of vasopressors*** |  |  |  |  |  | **X** |  |
| ***AEs and SAEs*** |  |  |  |  |  |  | **X** |

**7.2. Study time points and evaluation items**

**7.2.1. Screening**

(1) Participant characteristics

Age, sex, height, weight, medical history, comorbidities, medications (antihypertensive drugs, sleep-inducing drugs, antipsychotics, and central nervous system agents), ASA physical status, clinical frailty scale, surgery procedure, laboratory values of the latest surgery (hemoglobin, creatinine, and serum albumin).

(2) Vital signs

Noninvasive blood pressure (NIBP) and pulse rate will be measured in the ward after obtaining informed consent for this research.

**7.2.2. Pre-induction of general anesthesia**

(1) Vital signs

Before starting preoxygenation, NIBP and heart rate were measured twice as baseline vital signs.

**7.2.3. From the administration of sedatives to loss of consciousness**

(1) Vital signs

Blood pressure and heart rate will be measured at 1-min intervals.

(2) Time from the beginning of sedative drug administration to loss of consciousness.

(3) Injection pain

Participants will be asked a question about pain on injection after the start of sedative drug administration.

**7.2.4. From loss of consciousness to tracheal intubation**

(1) Vital signs

Blood pressure and heart rate will be measured at 1-min intervals.

**7.2.5. From the success of intubation to 3 minutes after intubation**

(1) Vital signs

Blood pressure and heart rate will be measured at 1-min intervals.

(2) Drug dose for induction of anesthesia

The number or dose of using any type of vasopressor between the start of sedative administration and 3 min after tracheal intubation

The dose of the trial drug for induction

**7.2.6. The day after surgery**

Occurrence of AEs

**7.2.7. Discontinuance**

1) Before starting anesthesia induction

In the cases where 1), 2), 6), or 7) of “6.5.5.1. Discontinuation criteria” will be applied.

Since the test drug will not be administered, no items to be observed will occur.

2) From induction of anesthesia to the analysis period

AEs will be monitored during anesthesia, which is routine practice, and by general postoperative examination, which is routine clinical practice.

**7.3. Evaluation method**

Not applicable.

**8. Outcomes**

**8.1. Primary outcome**

The primary outcome will be the incidence of hypotension between the start of propofol or remimazolam and 3 min after tracheal intubation. The definition of hypotension is an MBP <65 mmHg measured by NIBP.

**8.2. Secondary outcome**

- - 1. Maximum MBP 3 min after tracheal intubation
    2. Minimum heart rate after loss of consciousness.
    3. The number or dose of any type of vasopressor between the start of sedative administration and 3 min after tracheal intubation
    4. The time from the start of sedative drug administration to loss of consciousness. The loss of consciousness is defined as the loss of both the response to verbal stimuli and the reflex of the eyelashes. Loss of response to verbal stimuli was confirmed by calling his/her name every 10 s from the start of sedative drug administration.
    5. The dose of drugs used for anesthesia induction (*i.e.*, propofol or remimazolam)
    6. Incidence of injection pain. Participants will be asked a question about pain on injection after the start of sedative drug administration.

Pain on injection will be estimated by the following four-point scale:

0 = no pain

1 = mild pain (pain reported only in response to questioning and without any behavioral signs)

2 = moderate pain (pain reported in response to questioning and accompanied by a behavioral sign, or pain reported spontaneously without questioning)

3 = severe pain (strong vocal response or response accompanied by facial grimacing, drawing back the arm, or tears).

- - 1. Characteristics of the participants (age, sex, height, weight, medical history, comorbidity, medications [antihypertensive drugs, sleep-inducing drugs, antipsychotics, and central nervous system agents], ASA physical status, clinical frailty scale, surgery procedure, laboratory values of the latest surgery [hemoglobin, creatinine, and serum albumin]).
    2. Exploring factors associated with the incidence of hypotension

**8.3. Safety outcome**

AEs.

**9. Statistical analysis**

**9.1. Definition of an analysis set**

**9.1.1 Full analysis set (FAS)**

The full analysis set included all enrolled participants, administered the test or control drug, and obtained outcome data.

**9.1.2. Per protocol set (PPS)**

PPS is defined as participants excluded from FAS that have the following serious violations of the rules of the research protocol: selection criteria, exclusion criteria, concomitantly banned drugs, or concomitant therapy.

**9.1.3. Safety analysis set (SAS)**

The safety analysis set included all participants who will be enrolled, administered the test or control drug.

**9.2. Calculation of sample size**

The frequency of hypotension associated with the induction of general anesthesia was estimated to be 40% based on cases similar to our protocol extracted from our institutional data for a randomly selected month. In a previous study, the relative risk of remimazolam to propofol for the frequency of hypotension associated with induction of anesthesia was reported to be 0.28. If Fisher’s exact probability test with a type I error set at 5% and a power greater than 80% will be performed, it was estimated that at least 84 participants per group would be needed. Considering a dropout, we decided to include 45 participants in each group (*i.e.*, 90 in total).

**9.3. Statistical methods**

**9.3.1. Analysis for primary outcome**

**9.3.1.1. Main analysis**

The incidence of hypotension in the intervention and control groups will be calculated. The odds ratio (OR) with 95% confidence intervals (CIs) between both groups was calculated. The incidence of hypotension between the two groups will be compared using the Mantel−Haenszel χ^2^ test and adjusted OR for the stratification factor for randomization. Homogeneity across strata will be assessed using the Breslow-Day test.

**9.3.1.2. Additional analysis for primary outcome**

**1) Subgroups for analysis:** The subgroup for analysis will be generated as the following factors:

- 1. Charlson comorbidity index ≤2 or ≥3
  2. Clinical frailty scale ≤4 or ≥5
  3. Hypertension in the ward the day before surgery is defined as SBP ≥140 mmHg or diastolic blood pressure ≥90 mmHg
  4. Age (<90 or ≥90 years old)

The proportion of people aged ≥90 years in the total Japanese population is 2.1% (approximately 2.61 million), which is significantly different from the 12.8% (approximately 16.19 million) of people in their 80s. This was determined based on the assumption that this group is particularly affected by adverse drug reactions and circulatory instability.

**2) Sensitivity analysis of the primary analysis:** The OR with 95% CI adjusted for preinduction blood pressure (SBP ≥160 mmHg or <160 mmHg) will be calculated using the Mantel−Haenszel χ^2^ test. This cut-off point for SBP was as follows: (1) calculated based on the median value in the historical data of our institution for similar age groups; (2) and was determined regarding the exclusion criteria for blood pressure in a previous study on the efficacy and safety of remimazolam.

**3) The absolute risk reduction values with 95% CI** adjusted for stratification will be calculated using Mantel−Haenszel risk differences.

**9.3.2. Analysis for secondary outcome**

The incidence of injection pain for each group will appear as a relative risk with 95% CIs and will be compared using Fisher’s exact test.

Maximum blood pressure after tracheal intubation, the number of vasopressors used, the dose of drugs used for anesthesia induction, and the time from the start of sedative drug administration to loss of consciousness will appear median with interquartile ranges and will be tested using Student’s t-test or Mann–Whitney U test.

Logistic regression analysis will be performed using body mass index, sex, age, ASA physical status, Charlson comorbidity index, clinical frailty scale, preinduction hypertension, hypertension in the ward, use of antihypertensive drugs, use of sleep-inducing drugs, serum albumin level, and types of anesthesia induction drugs to explore the factors associated with the incidence of hypotension during the induction of general anesthesia.

**9.3.3. Safety analysis**

The incidence, type, and severity of AEs were summarized for each group.

**9.4. Interim analysis**

In this study, no interim analyses will be planned.

**10. Handling of AEs**

**10.1. Definition of AEs**

AEs are defined as all unfavorable or unintended medical events that occurred in the participants in the study (including unintended signs, clinically significant changes in laboratory values, symptoms, and worsening complications), whether causally related to the research protocol. In this study, events that occurred between the start of general anesthesia induction and the end of the follow-up period will be treated as AEs, and data on serious AEs and grade 2 or higher AEs will be recorded.

**10.2. Evaluation of AEs**

The degree of AEs will be evaluated according to the Common Terminology Criteria for Adverse Event v4.0.

**Grade 1**, Mild (no need for intervention for AEs)

**Grade 2**, Moderate (AEs require intervention such as outpatient drug treatment)

**Grade 3**, Severe (AEs require intervention such as inpatient treatment)

**Grade 4**, Life-threatening or incapacitated

**Grade 5**, Death

The relationship with the study drug is evaluated as follows:

**Related:** The causal relationship with the protocol of this study is valid and may not be due to exacerbations of primary disease, complications, or other treatments.

**Suspected:** The causal relationship with the protocol is unclear. This can also be explained by the exacerbation of the primary disease, complications, and other treatments.

**Not relevant:** It is not causally related to the protocol and can be clearly explained by exacerbations of the primary disease, complications, and other treatments.

**Definition of serious AEs**

1. Results in death
2. Life-threatening
3. Requires or prolongs patient hospitalization
4. Permanent or significant disability or incapacity
5. Congenital anomaly or birth defect
6. Other medically important conditions (*i.e.*, requiring intervention to prevent one of the previously listed outcomes)

**10.3. Anticipated AEs, etc.**

**Remimazolam:** In a study of Japanese patients who underwent surgery under general anesthesia, the incidence of adverse drug reactions in the remimazolam 12 mg/kg/h group was 42.7% (64/150 patients).

Major side effects: Hypotension 24.0% (36/150 cases), vomiting 7.3% (11/150 cases), nausea 6.7% (10/150 cases).

Serious side effects: Dependence (frequency unknown), bradycardia (4.7%), hypotension (26%), respiratory depression (frequency unknown), delayed arousal (frequency unknown), shock, anaphylaxis (frequency unknown).

Other side effects include: skin erythema (1−10%), headache (1−10%), delirium/dyskinesia (<1%), agitation (frequency unknown), second-degree atrioventricular block, ventricular extrasystoles, elevated blood pressure/hypertension (<1%), nausea/vomiting (1% to 10%), excessive drooling (<1%), elevated blood bilirubin (<1%), chills (1−10%), prolonged drug effect (frequency unknown).

**10.4. Measures for AEs**

**10.4.1. Treatment for research participants**

If a research participant suffers from AEs due to participation in the trial, the investigators will provide appropriate medical treatment and other necessary measures. If the condition requires treatment, the participant is informed of the condition. If the adverse event continues at the last observation point, the investigator will conduct follow-up studies until the condition recovers to the baseline value (Grade) or becomes clinically stable.

**10.4.2**. **Evaluation and recording**

Investigators should describe the name of the AE, date, severity, details of treatment or therapy, and outcome in the source document.

**10.4.3. Reporting of AEs**

The principal investigator shall report the AE to the head of the research institution, as necessary, following the institution's procedures.

**10.4.4. Reporting of SAEs**

If the principal investigator becomes aware of the occurrence of an SAE, he or she will promptly report it to the head of the research institution. The head of the research institution will take the necessary action promptly following the institution's procedures, listen to the opinions of the Ethics Review Committee, and take the necessary measures. The head of the research institution will also promptly report to the Minister of Health, Labor, and Welfare if the reported SAE is unpredictable and a direct causal relationship with this research cannot be ruled out. The head of the research institution will also disclose the status of the response and results to the Ethics Review Committee following the institution's procedures.

**11. Data management**

The case registration form and CRF are created using a paper form. These forms will be submitted to the research office, as described in the table below.

| Document type | Time of submitting | Means of submission |
| --- | --- | --- |
| Case registration form | At the time of enrollment | Direct delivery |
| Case report form | Within 7 days after surgery | Direct delivery |
| Study discontinuation | Within 7 days after surgery | Direct delivery |

**12. Effectiveness and safety evaluation committee**

In this study, no efficacy and safety evaluation committee will be established.

**13. Compliance, deviations, and changes to the research protocol**

**13.1. Compliance with the protocol**

Investigators must not deviate from or change the research protocol without prior written approval based on a review by the Ethics Review Committee.

**13.2. Deviation from the protocol**

1) Investigators may make deviations or changes from the research protocol before obtaining prior approval from the Ethics Review Committee if deviations or changes are unavoidable for medical reasons. In such cases, the investigators shall promptly submit the details and reasons for the deviation or change, as well as the proposed changes to the research protocol, if necessary, to the Ethics Review Committee for approval and obtain permission from the head of the research institution.

2) In the event of any deviation, a record shall be made of all deviations, together with the reasons, and the principal investigator shall report them to the head of the research institution and keep a copy of the record of the deviation.

**13.3. Modifying or changing the study protocol**

**13.3.1. Modification procedure**

If it is necessary to revise the research protocol, the principal investigator will discuss the issues to be revised by the research investigator. The principal investigator submits a document describing the protocol modifications to the head of the research institution. After review and approval by the Ethics Review Committee, permission from the head of the research institution was obtained. If it becomes necessary to revise the research protocol according to the direction of the head of the research institution based on the opinion of the Ethics Review Committee, the principal investigator will follow the same procedure to make the changes.

**14. Ethical matter**

**14.1 Ethical regulations**

We adhered to the ethical principles of the Declaration of Helsinki. This study was conducted according to the ethical guidelines for medical and health research involving human subjects established by the Ministry of Health, Labor, and Welfare of Japan. All studies will be conducted according to this protocol.

**14.2 Handling of personal information**

When personal information is handled, all participants will be identified by individual registry numbers used to anonymize CRFs or other documents related to this research at the time of enrollment. This individual registry number consists of numbers or letters not related to information that can identify a specific individual. From the anonymized information, the principal investigator shall prepare a correspondence list containing the names and medical record IDs of the participants so that the participants can be identified as necessary and shall strictly store and manage the list so that it will not be leaked to outside parties.

**14.3. Anticipated benefits and disadvantages as a result of participation in the study**

**14.3.1. Anticipated benefits**

There will be no direct benefit to the research subjects from their participation. Participation in this study may contribute to future medical advances.

**14.3.2. Anticipated disadvantages**

One potential economic disadvantage is that participants cannot reject the difference in medical costs due to the difference in drug prices between the trial and control drugs.

The AEs that may occur are described in Section "10.3. Anticipated AEs, etc." In the event of an AE, the investigators will take appropriate action following Section "10.4. Measures for AEs."

In this study, it will be not possible to determine which drug the participants would use. However, in this study no placebo will be used and in routine practice no detailed explanation of the drugs used for general anesthesia used in each individual case will be provided, hence, the subjects will not be disadvantaged.

**14.4. Handling of research results (including incidental findings)**

If the information that may seriously affect the health of the participant will be obtained through the tests to be conducted, the investigators will explain the information and take appropriate measures such as treatment or care.

**15. Procedures for receiving informed consent**

The researchers explained to the subjects the following information using a document approved by the ethics committee. After confirming that the patient fully understood the information, written consent was obtained for participation.

The items to be explained to the research subjects when receiving informed consent are, in principle, as follows:

1. Approval received to conduct the research
2. The name of the lead principal investigator and research institution
3. Objective and meaning
4. Methods, protocol, and duration of the study
5. Inclusion and exclusion criteria
6. Burden, risks, and benefits of participation
7. Withdrawal from the study
8. Subjects will not be disadvantaged
9. Other treatments
10. Viewing authority for data or information of participants
11. Handling of personal information
12. Storage and disposal of the data
13. Conflict of interest
14. Consultation with research participants and their related persons
15. The expense for this study
16. Medical care after this research (not applicable for this study)
17. Policy for incidental findings
18. Compensation for health damage
19. Possibility of reusing the data obtained in this study for a future study
20. Browsing data and information by people associated with research
21. Publication of research results
22. Policy for intellectual property rights

**15.1. Response to research participants and their related persons**

The principal investigator or subordinate investigators will respond to consultations from research participants and others.

**15.2. Obtaining informed consent from related persons of participants**

Not applicable.

**15.3. Informed assent**

Not applicable.

**16. Storage and disposal of data**

The principal investigator shall keep and manage the following documents and records related to this research in a locked cabinet with strict confidence. When storing electronic data, a password will be set and data stored on a computer or electromagnetic storage media such as a USB memory stick independent of the Internet and strictly stores and manages the data in a lockable cabinet when not in use. These documents and data related to this study should be kept until 5 years after the end of this study or 3 years after the publication of the results. After this period, the data will be completely deleted.

1. Research protocol
2. The explanatory document, consent form, and consent withdrawal form
3. Case registration form
4. Consent form with signature
5. CRF
6. List of map anonymized information of patients
7. Documents submitted to the Ethics Review Committee
8. Notification of the results of the Ethics Review Committee and notice of decision to give instructions by the head of the research organization
9. Other documents or records related to this research

**16.1. Secondary use of research data**

There is a possibility that the research data obtained in this study may be used for different research purposes or provided to other research institutions. In this case, a new research protocol will be prepared and approved by the Ethics Review Committee before implementation. Informed consent should be provided appropriately according to the content of the research.

**17. Research findings, conflicts of interest, and personal income for this study**

**17.1. Financial sources and relationships**

This study will be conducted with funds provided by the department of the research institution. No financial support from any particular company or organization was received. This study was conducted as medical research. It does not provide benefits to or for the convenience of a particular company or organization.

**17.2. Conflict of interest**

Researchers’ conflicts of interest shall be appropriately managed by the research organization following the policy and regulations of Yokohama City University. The status of the management of conflicts of interest and any changes in the details will be reported to the principal investigator.

**18. Cost burden and gratuities for research participants**

All medical expenses related to this study will be calculated based on the National Health Insurance of Japan. These expenses will be paid to the participants. There will be no additional financial burden on the research subjects as a result of participating in this research. No monetary or other rewards will be paid to the research participants in this study for participation or burden reduction.

**19. Compensation for health damage**

If a research participant suffers health damage due to this research, the investigators will provide the appropriate treatment and other necessary measures. In this case, treatment will be provided following the National Health Insurance, and the research participants will be responsible for their medical expenses.

The study will be conducted by administering the drug according to the information on the package insert. In case of death or disability due to side effects of medications, the participant is eligible to apply for relief benefits under the Medicines Adverse Reaction Relief System. Therefore, there was no financial compensation for health damage.

**20. Contents and method of the report to the director of the institute**

**20.1. Progress report of research**

The progress report of the research will be described once a year.

**20.2. End of the research**

At the end of this study, the research manager will report a summary of the results to the director of the institute.

**20.3. Early termination of research**

If the research is discontinued or interrupted, the research manager will report the situation to the director of the research institute. Specifically:

- - 1. If the safety or efficacy of the study is questioned, or if it is judged that the significance of the study will no longer exist.
    2. If it is judged that the successful execution of the study is difficult (*i.e.*, significant delay in case registration or many deviation events from protocol).

**21. Disclosure of research and publication of results**

**21.1. Registration of research**

This study will be registered with the University Hospital Medical Information Network Clinical Trials Registry [UMIN-CTR, http://www.umin.ac.jp/ctr/index-j.htm)] prior to implementation. It will be updated as appropriate according to changes in the research protocol and research progress. The results of this study will be registered when the study is completed.

**21.2. Disclosure of research results**

The results of this research will be presented at an international congress or in an academic journal. The first author and co-authors will be determined through discussions among those involved in the study. The presenter and the first author must review and approve the principal investigator before presentation and submission. The final publication will be reported to the head of the research institution.

**22. Monitoring and auditing**

**22.1. Monitoring**

Since this is a minor invasive study, no monitoring will be conducted. Self-inspection will be conducted every 3 months.

**22.2. Auditing**

Auditing will not be conducted.

**23. Attribution of intellectual property rights**

If intellectual property rights arise as a result of this research, these rights will belong to Yokohama City University.

**24. Organization of the research**

**24.1. Not applicable.**

**24.2. Research office**

Department of Anesthesiology and Critical Care Medicine, Yokohama City University Hospital

Address: 3-9, Fukuura, Kanazawa-ku, Yokohama, Kanagawa, Japan

Representatives of the secretariat: Masashi Yokose

**24.3. Principal investigator**

Department of Anesthesiology and Critical Care Medicine, Yokohama City University Hospital

Address: 3-9, Fukuura, Kanazawa-ku, Yokohama, Kanagawa, Japan

Research Supervisor: Masashi Yokose

**24.4. Statistical analysis manager**

Takahiro Mihara

Department of Health Data Science, Yokohama City University Graduate School of Health Data Science, Yokohama, Japan

Address: 3-9, Fukuura, Kanazawa-ku, Yokohama, Kanagawa, Japan

Phone: +8145872800

Statistical Advisor: Yusuke Saigusa

Department of Biostatistics, Yokohama City University Graduate School of Medicine, Yokohama, Japan

**24.5. Data management officer**

Hiroyuki Tanaka

Department of Anesthesiology and Critical Care Medicine, Yokohama City University Hospital

Address: 3-9, Fukuura, Kanazawa-ku, Yokohama, Kanagawa, Japan

Phone: +8145872800

**24.6. Outsourcing organization**

Not applicable.
