# Supplementary material for: Hypotension after general anesthesia induction using remimazolam in geriatric patients: Protocol for a double-blind randomized controlled trial
Source: PLoS One. 2022 Sep 30;17(9):e0275451. doi: 10.1371/journal.pone.0275451 (PMC9524631; doi:10.1371/journal.pone.0275451)
Supplement: S4 File — (DOCX) [file pone.0275451.s004.docx]

高齢者に対するプロポフォールを対照としたレミマゾラムの低血圧の発生頻度を検討する並行群間ランダム化比較

研究責任者：［所属］ 集中治療部 ［名前］ 横瀬　真志

版数：第1.1版

作成年月日：　2021年　4月　30日

改訂履歴

| 作成日 | 版数 |
| --- | --- |
| 2021年2月10日 | 第1.0版 |
|  |  |

内容

[**0. 概要** 6](#_Toc514065197)

[0.1. シェーマ 6](#_Toc514065198)

[0.2. 研究の目的と主要評価項目 6](#_Toc514065199)

[0.3. 研究対象者 6](#_Toc514065200)

[0.4. 治療 6](#_Toc514065201)

[0.5. 目標症例数 6](#_Toc514065202)

[0.6. 研究期間 6](#_Toc514065203)

[0.7. 研究責任者と問い合わせ先 7](#_Toc514065204)

[**1. 本研究の目的** 8](#_Toc514065205)

[**2. 背景と試験計画の科学的根拠** 8](#_Toc514065206)

[2.1. 対象疾患について 8](#_Toc514065207)

[2.2. 標準治療について 8](#_Toc514065208)

[2.3. 試験治療について 8](#_Toc514065209)

[2.4. 試験デザインと主要評価項目について 9](#_Toc514065210)

[2.5 本研究の意義 9](#_Toc514065211)

[**3. 薬物／機器情報** 10](#_Toc514065212)

[3.1. 試験薬（機器） 10](#_Toc514065213)

[3.2. 対照薬（機器） 10](#_Toc514065214)

[**4. 本研究で用いる規準・定義** 10](#_Toc514065215)

[**5. 研究対象者の選定方針** 10](#_Toc514065216)

[5.1. 選択基準 10](#_Toc514065217)

[5.2. 除外基準 11](#_Toc514065218)

[**6. 研究計画** 11](#_Toc514065219)

[6.1. 研究デザイン 11](#_Toc514065220)

[6.2. 目標症例数 11](#_Toc514065221)

[6.3. 研究期間 11](#_Toc514065222)

[6.4. 施設登録および症例登録・割付方法 11](#_Toc514065223)

[6.5. 治療計画 13](#_Toc514065224)

[**7. 観察・検査・調査・評価項目** 15](#_Toc514065225)

[7.1. スケジュール表 15](#_Toc514065226)

[7.2. 実施スケジュール時期と評価項目 15](#_Toc514065227)

[7.3. 評価の方法 16](#_Toc514065228)

[**8. 評価項目** 16](#_Toc514065229)

[8.1. 主要評価項目 16](#_Toc514065230)

[8.2. 副次評価項目 17](#_Toc514065231)

[**9. 統計解析** 18](#_Toc514065232)

[9.1. 解析対象集団 18](#_Toc514065233)

[9.2. 目標症例数の設定根拠 18](#_Toc514065234)

[9.3. 統計解析方法 18](#_Toc514065235)

[9.4. 中間解析 20](#_Toc514065236)

[**10. 有害事象の取り扱いについて** 20](#_Toc514065237)

[10.1. 有害事象の定義 20](#_Toc514065238)

[10.2. 有害事象の評価 20](#_Toc514065239)

[10.3. 予期される有害事象等 21](#_Toc514065240)

[10.4. 有害事象が発現した場合の措置 21](#_Toc514065241)

[**11. データマネージメント** 22](#_Toc514065242)

[**12. 効果安全性評価委員会** 22](#_Toc514065243)

[**13. 研究実施計画書の遵守，逸脱及び変更について** 22](#_Toc514065244)

[13.1. 研究実施計画書の遵守 22](#_Toc514065245)

[13.2. 研究実施計画書からの逸脱 22](#_Toc514065246)

[13.3. 研究実施計画書の変更 22](#_Toc514065247)

[**14. 倫理的事項** 23](#_Toc514065248)

[14.1 遵守すべき諸規則 23](#_Toc514065249)

[14.2. 個人情報等の取扱い 23](#_Toc514065250)

[14.3. 研究参加に伴い研究対象者に予測される利益及び不利益等 23](#_Toc514065251)

[14.4. 研究対象者に係る研究結果（偶発的所見を含む．）の取扱い 23](#_Toc514065252)

[**15. インフォームド・コンセントを受ける手続** 24](#_Toc514065253)

[15.1. 研究対象者等及びその関係者からの相談等への対応 24](#_Toc514065254)

[15.2. 代諾者等からインフォームド・コンセントを受ける場合 24](#_Toc514065255)

[15.3. インフォームド・アセントを得る場合 24](#_Toc514065256)

[15.4. 指針第12の6の規定による研究を実施しようとする場合 24](#_Toc514065257)

[**16. 試料・情報の保管及び廃棄の方法** 25](#_Toc514065258)

[16.1. 試料・情報の二次利用について 25](#_Toc514065259)

[16.2. 試料・情報のバイオバンクとしての利用 25](#_Toc514065260)

[**17. 研究の資金源等，研究機関の研究に係る利益相反及び個人の収益等，研究者等の研究に係る利益相反に関する状況** 25](#_Toc514065261)

[17.1. 資金源及び財政上の関係 25](#_Toc514065262)

[17.2. 利益相反 25](#_Toc514065263)

[**18.　研究対象者の費用負担・謝礼について** 26](#_Toc514065264)

[**19. 健康被害に対する補償** 26](#_Toc514065265)

[**20. 研究機関の長への報告内容及び方法** 26](#_Toc514065266)

[20.1. 研究の経過報告 26](#_Toc514065267)

[20.2. 研究の終了 26](#_Toc514065268)

[20.3. 研究の早期中止 26](#_Toc514065269)

[**21. 研究の情報公開及び結果公表** 26](#_Toc514065270)

[21.1. 研究の登録 26](#_Toc514065271)

[21.2. 研究結果の公表 26](#_Toc514065272)

[**22. モニタリング及び監査** 27](#_Toc514065273)

[22.1. モニタリング 27](#_Toc514065274)

[22.2. 監査 27](#_Toc514065275)

[**23．研究成果の帰属（知的財産権）** 27](#_Toc514065276)

[**24. 研究の実施体制** 27](#_Toc514065277)

[24.1. 研究代表者 27](#_Toc514065278)

[24.2. 研究事務局 27](#_Toc514065279)

[24.3. 参加施設および施設研究責任医師 27](#_Toc514065280)

[24.4. 統計解析責任者 27](#_Toc514065281)

[24.5. データマネージメント責任者 27](#_Toc514065282)

[24.6. 外部委託機関 28](#_Toc514065283)

[**25. 文献** 28](#_Toc514065284)

[**26. 付録** 28](#_Toc514065285)

**略語表**

| 略語 | 正式名称 | 説明 |
| --- | --- | --- |
| AE | Adverse event | 有害事象 |
| ASA | American Society of Anesthesiologists | アメリカ麻酔科学会 |
| ADL | Activities of Daily Living | 日常生活動作 |
| BMI | Body Mass Index | 体格指数 |
| CFS | Clinical Frailty Scale | 臨床的フレイル・スケール |
| CRF | clinical report form | 症例報告書 |
| FAS | full analysis set | 最大解析対象集団 |
| GABA | gamma-aminobutyric acid | γ-アミノ酪酸 |
| IADL | Instrumental Activities of Daily Living | 手段的日常生活動作 |
| PPS | Per Protocol Set of Subject | 研究実施計画書適合例 |
| PS | Performance status | 全身状態 |
| SAS | Safety Analysis Set of Subject | 安全性解析対象例 |
|  |  |  |

# **0. 概要**

## 0.1. シェーマ

80歳以上の全身麻酔単独で手術を受けるASAリスク1-3の患者

説明

同意取得

登録，ランダム割付

調整因子：高血圧

試験治療群（レミマゾラム群）

麻酔導入量として持続投与（12mg/kg/時）し

入眠が得られたところで投与終了

対照群（プロポフォール群）

麻酔導入量として持続投与（0.25mg/kg/10秒）し

入眠が得られたところで投与終了

主要評価項目：

麻酔導入薬投与開始後から気管挿管終了3分後までの間の低血圧の発生頻度

（低血圧の定義は平均血圧65mmHg未満）

## 0.2. 研究の目的と主要評価項目

目的：高齢者では全身麻酔導入薬のプロポフォールに伴う低血圧が生じやすい。重度低血圧は短時間であっても臓器障害や死亡率などのアウトカムとの関連性が指摘されている。短時間作動薬の新しいベンゾジアゼピン系薬であるレミマゾラム（アネレム®）は承認前治験においてプロポフォールよりも低血圧の発生頻度が少ない可能性が示されているが、80歳以上の超高齢者群においても、低血圧発生頻度が少ないかどうかは明らかでない。本研究の目的は80歳以上の高齢者群でレミマゾラムおよびプロポフォール使用に伴った低血圧の発生頻度を比較する事である。

主要評価項目：麻酔導入薬投与開始後から3分後までの間の低血圧の発生頻度

低血圧の定義は非観血的動脈圧（マンシェットを用いた上腕動脈での測定）で平均血圧65mmHg未満とする。

## 0.3. 研究対象者

80歳以上の全身麻酔単独で手術を受けるASAリスク1-3の患者

## 0.4. 治療

対照群：プロポフォールを持続投与（0.25㎎/kg/10秒）し、入眠が得られるところまで経静脈投与

試験治療群：レミマゾラム（アネレム®）を持続投与（12mg/kg/時）し、入眠が得られるところまで経静脈投与

## 0.5. 目標症例数

予定症例数：90例（各群45症例ずつ）

## 0.6. 研究期間

研究期間：実施許可日から2025年3月31日まで（4年間）

## 0.7. 研究責任者と問い合わせ先

所属：集中治療部　　　氏名：横瀬　真志

問い合わせ先：横浜市金沢区福浦3-9　横浜市立大学附属病院　麻酔科

# **1. 本研究の目的**

80歳以上の全身麻酔を受ける高齢者群において新しい全身麻酔導入薬であるレミマゾラムの麻酔導入時の血圧発生頻度を、プロポフォール使用群を対象とし比較する。

# **2. 背景と試験計画の科学的根拠**

## 2.1. 対象疾患について

本邦では老年人口の増加に伴い、高齢者が手術を受ける機会が増加している。高齢者は加齢による身体機能および予備力の低下や、多くの併存疾患を有することから、若年者と比較すると高い周術期合併症リスクがあるため慎重な管理を要する。実際、日本麻酔科学会による麻酔関連偶発症例調査(1)によると、調査対象となった2018年における心停止、高度低血圧の発生頻度は1か月未満の新生児期を除くと他の年代と比較して80歳代から上昇し始め90歳以上で最も多くなることが報告されている。

高齢者では生理学的身体的変化に起因する麻酔中の低血圧発生頻度が他の年代と比較して高く、年齢は全身麻酔導入後20分間の低血圧発生頻度と関連する（2）。また、全身麻酔中の低血圧は例え短時間であっても臓器障害や死亡率といった悪いアウトカムと関連すること（3）が近年指摘されてきている。このような背景から、特に高齢者に焦点を絞り低血圧予防の方策を確立することは臨床的に有用性があり、低血圧に伴う合併症の発生低下を介してより良い医療の提供と医療資源の削減が期待できる。

## 2.2. 標準治療について

全身麻酔導入薬としては現在プロポフォールが広く一般的に使用されている。これは、投与後の速い入眠と少ない副作用（気道過敏性・アレルギー等）というプロポフォールの特徴によるものである。しかしながら、血管拡張作用や心筋収縮抑制作用などに起因する低血圧の発生が臨床使用上のデメリットとなっている。特に高齢者では自律神経系の機能低下や動脈硬化など加齢に伴った生理学的身体的変化に起因する低血圧の発生頻度が他の年代と比較して高いと言われている（4, 5）。プロポフォールの添付文書上ではASAリスク分類^＊^Ⅲ・Ⅳの患者及び高齢患者での使用は慎重投与となっており、導入時の投与速度を通常量の1/2すなわち0.25mg/kg/10秒程度に減速することを推奨している。

＊ASAリスク分類 (ASA; American Society of Anesthesiologists)

Class I：正常健康患者

Class II：軽度の全身性疾患，軽度の糖尿病，高血圧，慢性気管支炎など，高齢者（70歳以上）．

新生児，肥満者も含む

Class III：中～高度の全身性疾患を有し，日常の活動が制限されている患者

Class IV：生命を脅かす程の全身性疾患を有し，日常の活動が不能である患者

Class V：手術の施行，非施行にかかわらず，24時間以上は延命できそうにない瀕死の患者

全身麻酔導入に伴う低血圧が持続する時間は一般的には短時間でありアウトカムへの影響も限定的と想像されがちであるが、全身麻酔中の低血圧は例え短時間であっても臓器障害や死亡率といった悪いアウトカムと関連することが指摘されている（3）。今後さらに増加する高齢者患者において、全身麻酔導入に伴う低血圧リスクを軽減する方策を探求することは、重要な課題である。

## 2.3. 試験治療について

レミマゾラム（アネレム®）は、超短時間作用型ベンゾジアゼピン系の鎮静麻酔薬であり、麻酔導入および麻酔維持を目的に承認された薬剤である。レミマゾラムはGABA_A_受容体のベンゾジアゼピン結合部位を介して作用を発現する。肝臓の組織エステラーゼによって速やかに代謝され、代謝物は活性を有していないことが短い時間作用の理由となる。全身麻酔薬としての機能に関する有効率（術中覚醒・記憶の有無、鎮静作用に対する救済処置の有無、体動の有無すべて「無」が有効）をアウトカムとした国内後期第Ⅱ相/第Ⅲ相臨床試験ではプロポフォールに対する非劣性が検証された（6）。同臨床試験の安全性解析対象集団における安全性評価項目ではプロポフォール群での処置を必要とする血圧低下の発生頻度が60.0%であったのに対して、レミマゾラム群では33.3%と低い傾向があることが示された。またレミマゾラムは承認前国内一般臨床試験における安全性解析対象集団における安全性評価項目で65歳以上の患者を多く含むASAリスク分類Ⅲ以上の重症患者における低血圧の発生頻度は41.9%と報告されているが（7）、主要評価項目として全身麻酔導入時に発生する麻酔薬に関連する低血圧の発生頻度について検証を行った研究はない。

レミマゾラムの重大な副作用としては依存性（頻度不明）、徐脈（4.7%）、低血圧（2.6%）、呼吸抑制（頻度不明、覚醒遅延（頻度不明）、ショック（頻度不明）、アナフィラキシー（頻度不明）などが添付文書上記載されているが、これらはプロポフォールでも起こり得るものであり、リスクについての優劣はない。また、上記の国内臨床試験では安全性評価も行われており、レミマゾラム投与によるリスクはプロポフォールと変わりないことが報告されている（6）。

レミマゾラムは全身麻酔導入薬として使用する。使用量は添付文書の記載に則り、シリンジポンプで持続投与（12mg/kg/hr）を行い、入眠が確認できたところで投与を終了し吸入麻酔薬による麻酔維持（セボフルレン、吸入濃度1.5%）に変更する。

## 2.4. 試験デザインと主要評価項目について

・並行群間、二重盲検、ランダム化、優越性試験。

全身麻酔導入後の血圧低下に関連する因子はこれまでに多数報告されている。観察研究をベースにこれらの因子すべてを調整するために必要となる症例数は膨大となる。またレミマゾラムは使用開始されたばかりの新規薬剤であり観察研究ではレミマゾラムの使用がどの程度となるか予想は現時点でできないことから、実現可能性として上記のデザインが望ましい。

本研究では、麻酔担当医師及び評価者を盲検化することは必要不可欠であると考えている。理由は、恣意的な脱落や先入観の入った評価が生じる可能性があるためである。患者の盲検化は薬剤名を口頭で発声することによって麻酔担当医師および評価者の盲検化が突破されることを防ぐ目的で行う必要性がある。患者に使用する薬物名を開示しないことは本研究における介入の侵襲性を高めることにもつながらない。

　　　　・主要評価項目

　　　　　麻酔導入薬投与開始後から3分後までの間の低血圧発生頻度

　　　　　低血圧の定義は非観血的動脈圧（マンシェットを用いた上腕動脈での測定）で平均動脈血圧65mmHg未満とする。

　　　　　本研究で平均血圧を評価項目としたのは、平均血圧が臓器還流を推察する際の指標として収縮期血圧よりも優れているためである。

　　全身麻酔中において、平均動脈圧65mmHg未満が5分以上持続することは、周術期の心臓、腎臓といった　臓器障害の発生や周術期死亡率と関連することが示唆されている (3)。また集中治療領域においても、敗血症治療ガイドラインでは臓器血流を維持する目的で平均血圧≧65mmHgを目標に血管収縮薬を用いることが推奨されている(8)。

## 2.5 本研究の意義

本邦では老年人口の増加に伴い、高齢者が手術を受ける機会が増加している。高齢者は加齢による身体機能および予備力の低下や、多くの併存疾患が存在することから、高い周術期合併症リスクがあり慎重な管理を要する。高齢者では生理学的身体的変化に起因する麻酔中の低血圧発生頻度が他の年代と比較して高く、全身麻酔中の低血圧は例え短時間であっても悪いアウトカムと関連することが指摘されている（3）。現在一般的に用いられている全身麻酔導入薬であるプロポフォールは速い鎮静作用と少ない副作用という特徴を持つが、その血管拡張作用や心筋収縮抑制作用から低血圧を引き起こし特に高齢者ではそのリスクが高い（4,5）。このような背景から、特に高齢者におけるレミマゾラムの低血圧発生頻度を比較検証することは臨床的に有用性があり、低血圧に伴う合併症の発生低下を介してより良い医療の提供と医療資源の削減が期待できる。

Negative resultsとなった場合には、新薬の登場によってもこれまでの臨床プラクティスを変更する必要性が低いことが示される。

# **3. 薬物／機器情報**

## 3.1. 試験薬（機器）

商品名：アネレム

一般名：レミマゾラムべシル酸

製造販売会社名：ムンディファーマ株式会社

効能・効果：全身麻酔の導入及び維持

用法・用量（使用方法）：通常、成人にはレミマゾラムとして12㎎/㎏/時の速度で、患者の全身状態を観察しながら、意識消失が得られるまで静脈内へ持続注入する。高齢者では適宜減量することが望ましい。

剤型（外観）：注射剤（バイアル）

## 3.2. 対照薬（機器）

一般名：プロポフォール

効能・効果：全身麻酔の導入及び維持

用法・用量：通常、成人には本剤を0.05mL/kg/10秒（プロポフォールとして0.5mg/kg/10秒）の速度で、患者の全身状態を観察しながら、就眠が得られるまで静脈内に投与する。なお、ＡＳＡIII及びIVの患者には、より緩徐に投与する。通常、成人には本剤0.20〜0.25mL/kg（プロポフォールとして2.0〜2.5mg/kg）で就眠が得られる。高齢者においては、より少量で就眠が得られる場合がある。就眠後は必要に応じて適宜追加投与する。

高齢者への投与：本剤は主に肝臓で代謝され、尿中に排泄される。一般に高齢者では、肝、腎機能及び圧受容体反射機能が低下していることが多く、循環器系等への副作用があらわれやすいので、投与速度を減速する（例えば、導入時の投与速度を約1/2すなわち本剤約0.025mL/kg/10秒に減速する）など患者の全身状態を観察しながら慎重に投与すること。

剤型（外観）：注射剤（アンプルもしくはバイアル）

# **4. 本研究で用いる規準・定義**

本研究は診断基準や病型分類を取り扱う内容ではないため、当てはまらない。

# **5. 研究対象者の選定方針**

## 5.1. 選択基準

(1)　80歳以上で定時手術を受ける患者

(2)　急速導入によるシングルルーメンチューブを用いた経口気管挿管で全身麻酔を施行される患者

(3)　全身麻酔導入前に区域麻酔を行わない患者

(4) 非心臓血管手術

(5) ASAリスク分類　Ⅰ～Ⅲ^＊^

＊ASA risk I：正常健康患者

ASA riskⅡ：軽度の全身性疾患，軽度の糖尿病，高血圧，慢性気管支炎など，高齢者（70歳以上）． 新生児、肥満者

ASA risk III：中～高度の全身性疾患を有し，日常の活動が制限されている患者

(6) 意思疎通および本人からの同意取得が可能な患者

## 5.2. 除外基準

(1) 未破裂脳動脈瘤・大動脈瘤の指摘がある患者

(2)　気道確保困難が予測されるもしくは既往がある患者

(3)　投与予定薬剤にアレルギー・禁忌がある患者

(4)　維持透析患者

(5)　重度の肝不全患者（Child-Pugh分類C）

(6)　未治療および不安定な虚血性心疾患患者

(7)　重度以上の大動脈弁・僧帽弁の弁膜症を有する患者

(8)　心房細動及び多発性の心房・心室性期外収縮等の不整脈を有する患者

(9)　うっ血性心不全患者（NYHA分類Ⅲ以上）

(10) 妊婦

(11)　BMI≧30

(12) 抗精神病薬および抗うつ薬を常用している患者

(13) その他、医師が研究参加に不適切と判断した患者

(14) 手術室入室後の麻酔開始前の平均血圧が70mmHg未満の場合　なお、この場合には症例登録後であっても、脱落及び中止には含めず、除外症例として扱う。

(15) 複数回の気管挿管が必要となった場合　なお、この場合には症例登録後であっても、脱落及び中止には含めず、除外症例として扱う。

# **6. 研究計画**

## 6.1. 研究デザイン

本研究は検証的、並行群間、二重盲検、標準治療対照、ランダム化比較試験である。

## 6.2. 目標症例数

研究全体の目標症例数：90例（試験治療群45症例，標準治療群45例）

症例数の設定根拠は「9.2.目標症例数の設定根拠」を参照。

## 6.3. 研究期間

研究期間：実施許可日から2025年3月31日まで（4年間）

登録期間：実施許可日から2022年9月30日

追跡期間：各症例において手術翌日まで

## 6.4. 施設登録および症例登録・割付方法

6.4.1. 登録センター

担当者：水野　祐介

施設名：横浜市立大学附属病院　麻酔科

TEL：045-787-2800

Mail：anesthes＠yokohama-cu.ac.jp

受付時間：平日8:30から17:15（土日，祝祭日，年末年始は受付けない）

6.4.2. 施設登録

本研究は単施設研究であるため、該当しない。

6.4.3. 症例登録および割付

（1）研究責任者又は研究分担者は、候補となる研究対象者から文書による同意を取得後、適格性を判断するために必要な情報を得るために問診や電子カルテの情報を確認する。研究対象者が選択基準を満たし、かつ除外基準のいずれにも該当していないことを確認する。

（2）研究責任者又は研究分担者は、症例登録票に必要事項を記載する。この際、研究対象者識別コードを付与するが、この研究対象者識別コードは単体で特定の個人を識別することができる情報を含まない任意のコードを用いる。

（3）症例登録票を登録センターに提出する。登録センターは症例登録票の記載内容を吟味し、適格であった場合には割り付けへ進む。

（4）研究期間中に一貫して患者リクルート、データ収集、麻酔管理に関与せずに薬剤投与のみを行う非盲検医師（以下、非盲検医師とする）は、薬剤割り付け表より試験薬を確定する。

（5）研究事務局は登録確認票を保存する。

6.4.4. 割付方法と割付調整因子

割り付けは麻酔準備開始前までに非盲検医師が行う。非盲検医師は1例目の症例登録開始前までに割り付け表を作成し、鍵のかかる保管庫で保管管理する。割り付け表の生成はコンピュータプログラムを元にランダムに生成された乱数を用いる。その鍵を管理することで解析終了までは麻酔担当医・評価者・データ解析者が確認することができないようにする。研究責任者または分担者は麻酔開始までに登録された被験者に関する情報を非盲検医師に連絡する。割付け結果は担当麻酔科医師および評価者には知られないようにする。

ランダム割り付けに際しては、高血圧の有無を割付調整因子とする層別ブロック割付を用いる。ランダム割付の詳細な手順はデータ収集を行う研究者に知らせない。

【割付調整因子の設定根拠】

・高血圧：

本研究の主要評価項目は8-1に記述のとおり平均血圧が65mmHg以下と設定した。普段の血圧が低い研究参加者はより多くの頻度で平均血圧65mmHg未満を満たす可能性があり、このような研究参加者の分布に差が生じた場合には研究結果をゆがめる可能性がある。血圧の閾値は入院後同意取得時に盲検化された医師が、病棟で被験者を仰臥位とし測定した非観血的動脈圧において収縮期血圧140mmHgもしくは拡張期血圧90mmHg以上のどちらかを満たした場合と定義する(9)。

6.4.5. 盲検化の方法

本研究では二重盲検（患者および評価者・麻酔担当医）を行うために試験薬と対象薬を投与するシリンジ、点滴ルート及び、点滴が刺入されている側の腕をタオルで覆うことで盲検化を図る。通常、麻酔導入に用いる薬剤の調剤は担当麻酔科医師が手術室内で患者入室前に行っている。本研究においては、薬剤の調剤および試験薬剤の投与を担当する医師（非盲検医師）を配置し、割付後に試験薬・対象薬の調剤・準備・投与を行う。この非盲検医師は薬剤の割付・薬剤投与に専従し、患者リクルートやデータ収集、また麻酔担当医師・評価者（盲検者）業務には関与しない。対象者の入室後に末梢静脈ルートの確保および、薬剤投与のセッティングを含めた麻酔導入の準備をこの非盲検医師が行い、すべての準備工程が完了した時点で麻酔担当医師および評価者が手術室へ入室し研究（すなわち全身麻酔）を開始することで評価者の盲検化を図る。

6.4.6. 開鍵（キーオープン）の必要性の判断及び手順

麻酔導入に用いる投与量から考えられる試験薬剤ならびに標準薬剤における特有の副作用はなく、有害事象が生じた際に行われる治療において薬剤の種類の違いに伴う特異的な治療法が必要となる可能性は低いと考えられる。このような理由から以下の場合に限りキーオープンを行う。

・緊急報告すべき重篤な事象（予測できない事象であり，因果関係が疑われる）と判断された場合

研究責任者への電話もしくは電子メールでの連絡の後に研究責任者が当該患者の割付を確認する。研究責任者は症例報告書にキーオープンとなった旨を理由および日時と共に記載する。データ固定までは統計解析担当者へは極力盲検性が保たれるよう、症例報告書の管理に注意する。

## 6.5. 治療計画

6.5.1. プロトコル治療

6.5.1.1. レミマゾラム投与群

レミマゾラムは全身麻酔導入薬として使用する。使用量は添付文書に記載された投与量に従い、シリンジポンプで持続投与（12mg/kg/時）を行い、入眠が確認できたところで持続投与を終了し、吸入麻酔薬（セボフルレン吸入濃度1.5%）による麻酔維持に変更する。入眠後3分間の用手換気を行った後にビデオ喉頭鏡による気管内挿管を行う。治療期間中は輸液ポンプを使用し、リンゲル液を600ml/時で持続投与を行う。

6.5.1.2. プロポフォール投与群

プロポフォールは全身麻酔導入薬として使用する。高齢者の投与に関する添付文書上の記載に従い、シリンジポンプで投与速度を0.25mg/kg/10秒に設定し持続静脈投与とする。入眠が確認できたところで持続投与を終了し、吸入麻酔薬（セボフルレン吸入濃度1.5%）による麻酔維持に変更する。プロポフォール投与速度は厚生労働省医政局、医薬品審査管理課に確認を行い承認の範囲内として差し支えないとの回答をいただいている。入眠後3分間の用手換気を行った後にビデオ喉頭鏡による気管内挿管を行う。治療期間中は輸液ポンプを使用し、リンゲル液を600ml/時で持続投与を行う。

6.5.2. 併用薬・併用療法

6.5.2.1. 併用薬・併用療法

・レミフェンタニル（麻薬性鎮痛薬）

麻酔導入薬開始3分前からシリンジポンプを用いて持続投与（投与速度は0.25mcg/kg/min）を行う。気管挿管後は0.05mcg/kg/minの投与速度とする。

・ロクロニウム（筋弛緩薬）

全身麻酔導入後、患者の入眠が確認された後に0.6mg/kgを静脈内投与する。

・セボフルレン（吸入麻酔薬）

患者の入眠確認後に1.5％の吸入濃度で投与を行う。

6.5.2.2. 併用禁止治療

プロトコル治療以外の全身麻酔導入目的に用いる鎮静薬

6.5.2.3. 併用可能治療

(1) 低血圧・徐脈に対する昇圧薬（エフェドリン、ネオシネジン、アトロピン）の投与

　　【昇圧薬投与プロトコル】

・平均血圧が65mmHgを下回った場合

その時の心拍数が80回/分未満の場合　エフェドリン4㎎を静脈投与する。

その時の心拍数が80回/分以上の場合　ネオシネジン0.05㎎を静脈投与する。

　　　　　　・心拍数45回/分未満の徐脈が持続する場合（著明な低血圧のない場合）

アトロピン0.5㎎を静脈投与する．持続する場合にはもう一度0.5㎎（1回目と合わせて計1.0㎎）までの投与を許容する。

　　　　　　・心拍数45回/分未満の徐脈に低血圧を併発している場合にはエフェドリン4㎎の静脈投与で代替することも可能とする。血圧、心拍数の推移を見て行われた昇圧薬の追加投与も許容する。

(2) その他，合併症および有害事象に対する対症療法

6.5.3. 減量・休薬基準

有害事象の発現により投与継続が困難であると担当医師等が判断した場合はプロトコル治療を中止する。減量・休薬基準は設定しない。

6.5.4. 増量・再開基準

増量・再開基準は設定しない。

6.5.5. 個々の研究対象者における研究の中止

6.5.5.1. 中止基準

以下の基準のいずれかに該当する場合は，研究対象者の研究を中止する。

（1）患者または代諾者から同意撤回の申し出があった場合

（2）登録後に選択基準に合致しない又は除外基準に抵触し対象として不適切であることが判明した場合

（3）疾患の症状（重篤なバイタルサイン変化の出現・収縮期血圧70mmHg未満が持続する昇圧薬への反応に乏しい血圧低下・心拍数40未満の高度徐脈が持続・手術キャンセル）により研究の継続が困難な場合

（4）有害事象の発現により研究の継続が困難な場合

（5）研究実施計画書からの重大な逸脱が発生した場合

（6）その他，研究の継続が好ましくないと研究責任者又は研究分担者が判断した場合

6.5.5.2. 中止手順

（1）研究責任者又は研究分担者は、研究期間中に研究対象者への試験薬の投与を中止する場合、必要な観察、検査及び評価を行う。当該研究対象者には麻酔覚醒後にその旨を説明する。

（2）研究責任者又は研究分担者は、有害事象の発現等，研究対象者の安全性の問題により当該研究対象者への試験薬の投与を中止した場合には研究対象者に対し適切な処置を施す。また、症状（臨床検査値も含む）が試験薬投与前もしくはベースラインの状態に回復するまで、又は症状が安定するまでの期間、経過観察を継続し、その転帰を記録する。

（3）投与を中止した理由を症例報告書に記載する。

6.5.5.3. プロトコル治療終了（中止）後の後治療

本研究では該当しない。

# **7. 観察・検査・調査・評価項目**

## 7.1. スケジュール表

## 7.2. 実施スケジュール時期と評価項目

7.2.1. スクリーニング期間

(1)研究対象者背景

・性別、年齢、身長、体重、既往歴、合併症、内服薬（降圧薬・睡眠薬・抗精神病薬、抗うつ薬以外の中枢神経系作用薬）、ASAリスク、Clinical Frailty Scale （CFS、 臨床的フレイル^＊^・スケール)　、手術術式

^＊^フレイル　［Frailty（虚弱）の日本語訳］　健康な状態と要介護状態の中間に位置し、身体的機能や認知機能の低下が見られる状態のこと。

・CFSのカテゴリー (10)

1．極めて健康：壮健、活動的、精力的、意欲十分で、一般的に規則的な運動をしており、この年代では最も元気である

2. 健康：特別な病気はないがカテゴリー1よりはやや劣る。

3．疾病を有するが、よくコントロールされている。

4．見かけ上やや弱い：依存してはいないが行動が遅く、疾病の症状がある。

5. 経度にフレイル：日常生活で手段的ADL（Instrumental Activities of Daily Living; IADL）について　　　　ある程度依存している。

6．中等度にフレイル：IADLのみならずADLにも支援が必要。

7．重度のフレイル：生活全般において完全に依存している。しかし、身体状態は安定しており、（半年以内の）死亡リスクは高くない。

8．非常に重度のフレイル：全介助であり、死期が近づいている（例：軽度の疾患でも回復しない）。

9．疾患の終末期：死期が近づいている。生命予後は半年未満だが、それ以外では明らかにフレイルとは言えない。

(2)バイタルサイン

・血圧、脈拍数（入院後の同意取得時に病棟で測定し記録する）

7.2.2. 麻酔導入前

（1）バイタルサイン

・血圧、心拍数；血圧を2回測定しそれぞれの血圧測定時の心拍数を記録する

7.2.3. 薬剤投与前から入眠まで

　　 (1)バイタルサイン

・血圧、心拍数；血圧は1分間隔で測定しそれぞれの血圧測定時の心拍数を記録する

(2)入眠までの時間

・投与開始から入眠までの時間を測定し記録する

　入眠の確認はまず10秒ごとの声掛けを行う。次に声掛けへの反応消失後は睫毛反射を確認する

　双方の消失をもって入眠と定義する

(3)注入時血管痛

・鎮静薬投与開始10秒後に本人へ血管痛の程度を確認し記録する

　（これは7.2.3.(2)の入眠までの時間を測定する際の初回声掛けを兼ねる。評価方法については8.2．に記載）

7.2.4. 入眠から気管挿管まで（3分間の用手換気）

(1)バイタルサイン

・血圧、心拍数；血圧は1分間隔で測定しそれぞれの血圧測定時の心拍数を記録する

7.2.5. 気管挿管から挿管後3分間

(1)バイタルサイン

・血圧、心拍数；血圧は1分間隔で測定しそれぞれの血圧測定時の心拍数を記録する

（2）鎮静薬・昇圧薬の使用量

　麻酔導入時に使用した鎮静薬および昇圧薬の投与量を確認し記録する

7.2.6. 手術翌日

　　　有害事象の発生の有無について術翌日に確認し記録する。

7.2.7.中止時

　　　本研究における中止は麻酔開始前、麻酔開始から解析期間の2つの状態が想定される。

　　　・麻酔開始前（6.5.5.1. 中止基準の1,2,6,7が該当）

　　　　試験薬の投与は行われていないため、特に観察する項目は発生しない。

　　　・麻酔開始から解析期間（中止基準3,4,5,6,7）

　　　　通常診療で行われている麻酔中のモニタリングおよび、術後に通常臨床で一般的に行われる診察によって有害事象の観察を行う。

## 7.3. 評価の方法

本研究では該当しない。

# **8. 評価項目**

## 8.1. 主要評価項目

麻酔導入薬投与開始後から気管挿管終了3分後までの間の低血圧の発生頻度

低血圧の定義は非観血的動脈圧（マンシェットを用いた上腕動脈での測定）で平均血圧65mmHg未満とする。

【設定根拠】

・平均血圧は臓器還流を推察する際の指標としてより適している。

・全身麻酔中において、平均動脈圧65mmHg未満が持続することは、周術期の心臓、腎臓といった臓器障害の発生や周術期死亡率の軽度から中等度のリスク上昇と関連することが示唆されている (3)。

・集中治療領域においては、敗血症治療ガイドラインでは臓器血流を維持する目的で平均血圧≧65mmHgを目標に血管収縮薬を用いることが推奨されている(8)。

## 8.2. 副次評価項目

(1) 挿管後の最高血圧

　気管挿管後から挿管3分後までの間に測定された非観血的平均動脈血圧で最高値と定義し記録する。

　根拠：個々の患者における気管挿管時の麻酔深度の指標として多くの研究で用いられている。

(2) 入眠後の最低心拍数

　入眠後から研究期間内までの間に心電図で測定された心拍数で最も低い値と定義し記録する。

　根拠：鎮静薬の心血管抑制作用による血圧低下と心拍数低下との関連が予想されるため記録する。

(3) 昇圧薬の使用回数

麻酔導入から挿管3分後までの間に使用した昇圧薬の使用回数を記録する。昇圧薬の種類は問わず、投与された回数のみを記録し評価する。

根拠：血圧低下の強度、持続の程度を評価する指標として多くの過去の類似研究で用いられている。

(4) 入眠までの時間

　鎮静薬投与開始から次に定義する入眠までの時間を測定する。

入眠は麻酔薬投与開始から10秒ごとに声掛けを行い、反応がなくなったところで睫毛反射の消失を確認する。両者の消失で意識消失とする。なお初回の声掛けは下記の「鎮静薬投与時の血管痛発生の頻度」における評価をもって代用する。

根拠：過去の類似研究では、意識消失の確認を行う間隔として5秒毎から30秒毎の幅で行われている。本研究では、入眠までの時間として得られるデータ量と実臨床上のプラクティスおよび実行の煩雑さなどを勘案し、上記の間隔での確認と設定した。

(5) 入眠までに必要とした試験治療薬及び対象治療薬の使用量

　シリンジポンプを用いて決められた速度で持続投与を行い、入眠時に投与を終了する。その時点までに使用した鎮静薬の投与量（mL）を記録する。

　根拠：実際に入眠が得られる投与量を先行研究との比較を目的とする。

(6) 鎮静薬投与時の血管痛発生の頻度

　注入開始 10秒後に「点滴の部分の痛みがありますか？」という声掛けによる聴取、もしくは対象者の行動を以下の4点スケール(11)で記録する。

**Scale: 0 =** 無痛

**Scale: 1** = 弱い痛み (質問に対する反応のみで、身体的な反応は認めない)

**Scale: 2** = 中等度の痛み (身体的な反応を伴った質問に対する反応、もしくは質問無しに自発的な対象者の発言)

**Scale 3** = 強い痛み (強い声の反応、顔をゆがめる、腕を引っ込める、流涙)

　　　　 (7) 患者背景　［年齢・性別・身長・体重、既往歴、合併症、内服薬（降圧薬・睡眠薬・抗精神病薬、抗うつ薬以外の中枢神経系作用薬）、ASAリスク、CFS］

(8)　低血圧発生頻度に影響を及ぼす患者要因の探索

## 8.3. 安全性評価項目

　　　　・有害事象

# **9. 統計解析**

## 9.1. 解析対象集団

解析対象集団は、以下のように定義する。主たる解析対象集団はFASとする。

9.1.1. 最大解析対象集団（full analysis set：FAS）

本研究に登録され、ランダム化後に試験薬を投与され、アウトカムデータが収集されたすべての研究対象者をFASとする。ただし、重大な研究実施計画書違反（同意未取得、契約期間外の登録等）の研究対象者については除外する。

9.1.2. 研究実施計画書に適合した対象集団（per protocol set：PPS）

FASから、研究方法や併用療法など研究実施計画書の規程に対して、以下の重大な違反があった症例を除いた集団をPPSとする。

・選択基準違反

・除外基準違反

・併用禁止薬違反

・併用禁止療法違反

9.1.3. 安全性解析対象集団（safety analysis set：SAS）

本研究に登録され、少なくとも１度は試験薬を投与された集団をSASとする。

## 9.2. 目標症例数の設定根拠

研究全体の目標症例数：90例 （試験薬群45例、対照群45例）

【設定根拠】

自施設の過去一ヵ月の全身麻酔のみで導入した75歳以上の症例において、今回の研究のプロトコルに類似した麻酔導入が行われた症例8例における低血圧の発生頻度は37.5%であった。本研究の対象は80歳以上であり、この結果よりも低血圧の発生頻度が高くなることが予想されることから、40%と見積もる。過去の研究（5）のレミマゾラムの全身麻酔導入時に限定した低血圧発生に関するリスク比はプロポフォールを対象とした場合で0.28であった。両側αエラー　0.05、検出力　0.8　両群の比を1:1としFisher正確確率検定を行うとした場合、1群当たりの症例数は42例、計84例と計算される。若干の脱落症例数を勘案し1群45例、計90例とした。

## 9.3. 統計解析方法

9.3.1. 主要評価項目の解析

9.3.1.1 主要な解析

各群における低血圧発生割合を算出し、そのオッズ比と95％信頼区間を算出する。試験群および対照群における低血圧発生割合は、割付調整因子を考慮しMantel-Haenszelχ2検定で群間比較を行い、調整オッズ比の推定を行い、Wald法で95%信頼区間を算出する。層別間の均質性はBreslow-Day検定で評価する。両側検定でp<0.05で統計学的に有意差があると判定する。

9.3.1.2. 副次的な解析

１）本研究では主要評価項目に対する副次的な解析として、以下の因子でサブグループ解析を行う。

・チャールソン併存疾患指数^*^（2011年改訂版）： 2点以下or 3点以上

・CFS： 4点以下 or 5点以上

・高血圧の有無: 病棟での血圧で評価

収縮期血圧140mmHg未満 or 140ｍｍHg以上もしくは拡張期血圧90ｍｍHg未満or 90mmHg以上 (9)

・年齢：90歳未満or 90歳以上

　　この区別を裏付ける明確な参考文献は存在しない。日本人の2017年の総務省統計局のデータによると、90歳以上が全人口に占める割合は2.1%（約261万人）であり、80歳代の12.8%（約1,619万人）と大きな相違があり、特に薬物の副作用や循環動態の不安定性による影響を強く受ける群と仮定して決定した。(12)

*チャールソン併存疾患指数；慢性疾患の合併に基づいて、患者の短期的な死亡リスクを予測するために開発された、加重スコアリングシステムである。次に示す表のようにいくつかの疾患カテゴリーごとに重みづけられた点数を加算していく（最小0点、最大24点）。スコアが高いほど併存疾患が多い状態であり、短期的な死亡リスクも点数の上昇に応じて上昇するとされている。 (13)


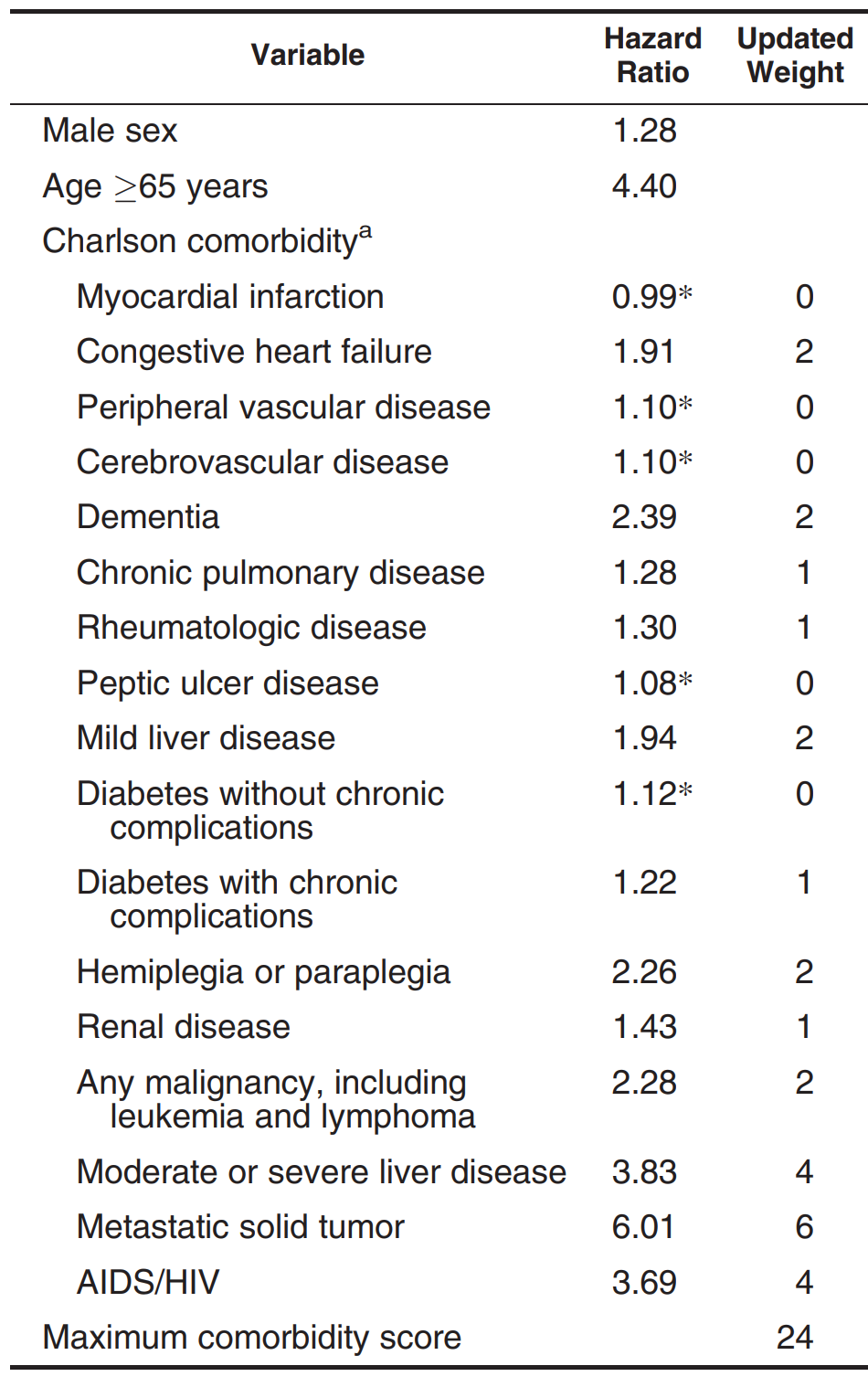


２）主要な解析の感度分析：　麻酔導入前血圧（収縮期血圧160ｍｍHg未満or以上）を考慮したMantel-Haenszelχ2検定で群間比較を行い、調整オッズ比および95%信頼区間を算出する。麻酔導入前血圧のカットオフ値は類似した年齢層の過去の自施設データにおける中央値および、レミマゾラムに関する国内Ⅱ相後期／Ⅲ相試験において収縮期160mmHg以上の高血圧患者が除外項目となっていることから、その値を基準とした(6)。

３）Mantel-Haenszel法による主要評価項目の調整リスク差および信頼区間の計算

9.3.2. 副次評価項目の解析

・各群における鎮静薬投与時の血管痛発生の割合

リスク比の点推定値とその95％信頼区間を算出する。

両群の割合を比較するため、Fisher正確確率検定を行う。

・挿管後の最高血圧、昇圧薬の使用回数、入眠までの時間、入眠までに必要とした試験治療薬及び対象治療薬の使用量

中央値と四分位範囲とを算出し、両群の比較を行うためStudent’s t検定もしくはMann-Whitney U検定を行う。両側検定でp<0.05で統計学的に有意差があると判定する。

・患者背景、術式

記述および集計を群ごとに行う。

　　　　・低血圧発生頻度に影響を及ぼす患者要因の探索

下記の因子を考慮したロジスティック回帰解析を行う。

　 BMI、性別、年齢、ASAリスク、チャールソン併存疾患指数、CFS、麻酔導入前高血圧、病棟高血圧、血清アルブミン値、降圧薬内服の有無、睡眠薬の使用、麻酔導入薬の種類

9.3.3. 安全性解析

有害事象発現率種類及び重症度を群ごとに集計・算出する。

## 9.4. 中間解析

本研究では中間解析は実施しない。

# **10. 有害事象の取り扱いについて**

## 10.1. 有害事象の定義

有害事象（Adverse Event：AE）とは、研究との因果関係の有無に関わらず研究対象者に生じた全ての好ましくない又は意図しない医療上の出来事であり、意図しない徴候、臨床検査値の臨床的に有意な変動、症状、合併症の悪化を含む。

本試験では全身麻酔開始から後観察期間終了までの間に発生した事象を有害事象として取り扱い、重篤な有害事象およびGrade2以上の有害事象についてのデータを収集する。

## 10.2. 有害事象の評価

AEの程度はCommon Terminology Criteria for Adverse Event v4.0に準じ、以下のように評価する。なお、実際治療したかどうかではなく必要性で判定する。

Grade1：軽度（AEに対して、治療介入の必要がない）

Grade2：中等度（AEに対して、外来薬物治療などの治療介入が必要）

Grade3：重度（AEに対して、入院での治療が必要）

Grade4：生命を脅かす、または活動不能となる

Grade5：死亡

プロトコル治療との因果関係は以下の3つに分類する

(1) 関連あり：プロトコル治療との因果関係は妥当で原疾患の増悪、合併症、他の治療などが原因でないと考えられる。

(2) 関連が否定できない：プロトコル治療との因果関係は明らかでない。原疾患の増悪、合併症、他の治療などでも説明しうる。

(3) 関連なし：プロトコル治療との因果関係がなく、原疾患の増悪、合併症、他の治療などで明らかに説明できる。

以下のいずれかに該当するものを「重篤なAE」とする

(1) 死亡に至るもの

(2) 生命を脅かすもの

(3) 治療のため入院若しくは入院・加療期間の延長が必要となるもの

(4) 永続的または顕著な障害・機能不全に陥るもの

(5) 子孫に先天的異常をきたすもの

(6) その他医学的に重要な状態（1～5に至らずとも、至らぬよう処置を必要とするような重大な事象）

## 10.3. 予期される有害事象等

レミマゾラムについては以下の通りで、プロポフォールについては添付文章の記載の通りとする。日本人の全身麻酔施行手術患者を対象とした第II／III相実薬対照無作為化単盲検比較試験ではレミマゾラム12mg/kg/時投与群における副作用発現割合は42.7％（64／150例）であった．主な副作用は血圧低下24.0％（36／150例）、嘔吐7.3％（11／150例）、悪心6.7％（10／150例）であった。

重大な副作用：依存性（頻度不明）、徐脈（4.7％）、低血圧（26％）、呼吸抑制（頻度不明）、覚醒遅延（頻度不明）、ショック、アナフィラキシー（頻度不明）

その他の副作用：皮膚紅斑（１%以上10％未満）、頭痛（１%以上10％未満）、せん妄・ジスキネジー（1%未満）、激越（頻度不明）、第2度房室ブロック・心室性期外収縮・血圧上昇・高血圧（1%未満）、悪心・嘔吐（1%以上10%未満）、流涎過多（1%未満）、血中ビリルビン上昇（1%未満）、悪寒（1%以上10%未満）、薬効延長（頻度不明）

## 10.4. 有害事象が発現した場合の措置

10.4.1. 研究対象者への措置

研究責任者又は研究分担者は、有害事象が発生した場合、研究対象者の安全確保のため必要に応じ研究対象者に対し、治療及び試験薬投与の中止等、適切な措置を講じる。治療等が必要となった場合は、その旨を研究対象者に伝える。

研究責任者又研究分担者は、試験薬終了時の最終観察時点で有害事象が継続している場合は、それ以降もベースライン値の状態（Grade）に回復するまで、又は臨床的に安定するまで追跡調査を実施する。

10.4.2. 評価及び記録

研究責任者又は研究分担者は、発現した有害事象について、原資料（診療録等）に有害事象名、発現日、重篤・非重篤の別、処置・治療の内容、転帰（回復した場合は回復時期、症状が固定した場合はその時期）を記載する。

10.4.3. 有害事象の報告

有害事象の発生を知った場合は、研究責任者又は研究分担者は、症例報告書により、研究責任者（又は研究事務局）に報告する。研究責任者は、研究機関の手順書に従い、必要に応じて、研究機関の長等に当該有害事象を報告する。

10.4.4. 重篤な有害事象の報告

重篤な有害事象の発生を知った場合、研究責任者は速やかに研究機関の長に報告する。（第1報報告）第1報の報告書に当該重篤な有害事象に関する詳細な情報が不十分であった場合は、詳細な情報を同様の方法で研究機関の長に報告する。また、追加情報を入手した場合は、できるだけ速やかに同様の方法で研究機関の長に提出する。

研究機関の長は、研究責任者から重篤な有害事象の報告があった場合、施設の手順書に従って速やかに必要な対応を行うとともに、倫理審査委員会の意見を聴き、必要な措置を講じる。また研究機関の長は、報告された重篤な有害事象が予測できないものであり、かつ本研究と直接の因果関係が否定できない場合には、速やかに厚生労働大臣に報告するとともに、施設の手順書に従って、対応状況および倫理審査委員会の結果を公表する。

# **11. データマネージメント**

症例登録票と各症例報告書等は、紙媒体で作成し、下表に記載されたとおりに研究事務局へ提出する。

症例登録票および症例報告書等の提出時期と提出方法

| 種類 | 提出時期 | 提出手段 |
| --- | --- | --- |
| 症例登録票 | 症例登録時 | 直接手渡し |
| 症例報告書 | 術後7日以内 | 直接手渡し |
| 中止時 | 術後7日以内 |  |

# **12. 効果安全性評価委員会**

本研究では効果安全評価委員会は設置しない。

# **13. 研究実施計画書の遵守，逸脱及び変更について**

## 13.1. 研究実施計画書の遵守

研究責任者又は研究分担者は、倫理審査委員会の審査に基づく文書による事前の承認を得ることなく、研究実施計画書からの逸脱又は変更を行ってはならない。

## 13.2. 研究実施計画書からの逸脱

1）研究責任者又は研究分担者は、研究対象者の緊急の危険を回避するためのものである等医療上やむを得ない場合には、倫理審査委員会の事前の承認を得る前に、研究実施計画書からの逸脱あるいは変更を行うことができる。その際には、研究責任者又は研究分担者は、逸脱又は変更の内容及び理由並びに研究実施計画書の変更等が必要であればその案を速やかに、倫理審査委員会に提出してその承認を得るとともに、研究機関の長の許可を得る。

2）研究責任者又は研究分担者は、研究実施計画書からの逸脱があった場合には、逸脱事項についてその理由とともに全て記録を作成し、研究責任者は研究機関の長に報告しなければならない。研究責任者は、逸脱に関する記録の写しを保存する。

## 13.3. 研究実施計画書の変更

13.3.1. 研究実施計画書の変更手順

研究実施計画書の内容を変更する場合は、以下の手順により行う。

1）研究責任者は、研究実施計画書の変更が必要と判断した場合は、研究事務局及び分担者に研究実施計画書変更案及びその他必要な資料・情報について協議する。

2）研究責任者は、前項により協議された変更内容を記載した文書及び変更した研究実施計画書を研究機関の長に提出し、倫理審査委員会の審査を受け、倫理審査委員会の承認並びに研究機関の長の許可を取得する。

3）研究責任者は、倫理審査委員会の意見に基づく研究機関の長の指示により、研究実施計画書の修正等が必要となった場合は、同様の手順で変更を行う。

# **14. 倫理的事項**

## 14.1 遵守すべき諸規則

本研究は、ヘルシンキ宣言に基づいた倫理原則を遵守し、「人を対象とする医学系研究に関する倫理指針」に従って実施する。担当医師は、本プロトコルを遵守してプロトコル治療を実施する。

## 14.2. 個人情報等の取扱い

本研究では患者を登録する際に、研究対象者識別コードを付与する。研究対象者識別コードは、イニシャルやカルテID等のような特定の個人を識別できる情報とは無関係の数字記号等で構成され、症例登録票や症例報告書等の本研究に関する書類を作成する際には研究対象者識別コードを使用することで匿名化を行う。研究責任医師は、匿名化された情報から、必要に応じて研究対象者を識別することができるように研究対象者の氏名やカルテID等の情報が記載された対応表を作成し、外部に漏洩することがないよう厳重に保管管理を行う。（管理責任者：横瀬真志）

## 14.3. 研究参加に伴い研究対象者に予測される利益及び不利益等

14.3.1. 予想される利益

本研究参加により研究対象者に直接の利益は生じない。研究参加により将来の医療の進歩に貢献できる可能性がある。

14.3.2. 予想される不利益

本研究で用いる薬剤はいずれも本研究の対象に対する適応承認のうえで保険適用されているものであり、日常診療に比して、研究対象者が本研究に参加することで経済上の負担はない。二重盲検で生じうる経済的な不利益としては試験薬と対象薬の薬価の違いによる医療費の違いを拒否できない点がある（レミマゾラム・2218円/瓶、プロポフォール・400-700円/管・瓶）。実際の支払額は対象者の保険の種類や自己負担限度額制度等によって異なるが、後期高齢者医療制度に則った1割負担による単純計算において、おおよそ150-180円程度の差額が生じる。

本研究に参加する事で研究対象者に起こり得る有害事象は「10.3. 予期される有害事象等」に記載した。研究責任者又は研究分担者は、有害事象が発生した場合、「10.4. 有害事象が発現した場合の措置」に従い適切に対処する。 また二重盲検とすることによる不利益は、対象者がどちらの薬剤を用いられたかを知ることができない点があるが、研究デザイン上プラセボを用いることはなく、また日常臨床においても各々の全身麻酔薬の名前やその詳細について説明することはないため、「手術のための麻酔をかける」という意味においては対象者の不利益は生じない。

## 14.4. 研究対象者に係る研究結果（偶発的所見を含む。）の取扱い

本研究で研究対象者の健康、子孫に受け継がれ得る遺伝的特徴等に関する重要な知見が得られる可能性はないが、実施する検査等により、研究対象者の健康に重大な影響を与える情報（偶発的所見を含む）を入手した場合は、研究責任医師または分担医師が研究対象者に説明し、治療や処置を行う等の適切な措置を講じる。また、研究対象者個別における研究参加に伴う結果は診療の中で研究対象者本人に説明する。

# **15. インフォームド・コンセントを受ける手続**

研究責任医師および分担医師は、患者が研究に参加する前に、施設の倫理審査委員会で承認された同意・説明文書を用いて以下の事項を十分に説明する患者が内容を十分理解したことを確認した後、参加の同意を文書により取得する。

インフォームド・コンセントを受ける際に研究対象者等に対し説明すべき事項は、原則として以下のとおりとする。ただし、倫理審査委員会の意見を受けて研究機関の長が許可した事項については、この限りでない。

1 研究の許可を受けていること

2 研究機関及び研究責任者

3 研究の目的及び意義

4 研究の方法及び期間

5 研究対象者として選定された理由

6 負担並びに予測されるリスク及び利益

7 撤回できること

8 不利益を受けないこと

9 他の治療方法について

10 資料の閲覧

11 個人情報等の取り扱い

12 試料・情報の保管及び廃棄の方法

13 利益相反

14 研究対象者等及びその関係者からの相談

15 費用について

16 研究実施後の医療提供

17 偶発的所見の取り扱い

18 健康被害への補償

19 将来の研究の可能性

20 関係者が試料・情報を閲覧すること

21 研究成果の公表について

22 知的財産権について

## 15.1. 研究対象者等及びその関係者からの相談等への対応

研究対象者等やその関係者からの相談には，研究責任者または研究分担者が対応する。

## 15.2. 代諾者等からインフォームド・コンセントを受ける場合

本研究では満20歳以上かつ本研究への参加について患者本人から文書による同意を取得できる者を対象としているため該当しない。

## 15.3. インフォームド・アセントを得る場合

本研究では満20歳以上の患者を対象としているため該当しない。

## 15.4. 指針第12の6の規定による研究を実施しようとする場合

本研究では該当しない。

# **16. 試料・情報の保管及び廃棄の方法**

本研究では生体試料の保管は行わない。研究責任医師は、下記に掲げる本研究に関する文書および記録を施錠可能な保管庫で厳重に保管管理する。電子データで保管する場合は、パスワードを設定した上で、インターネットから独立したパソコンまたはUSBメモリ等の電磁的記録媒体にて保管し、使用していない時は施錠可能な保管庫で厳重に保管管理する。紙媒体のものも同様に施錠可能な保管庫で管理する。保管期間は、研究の終了について報告された日から5年間もしくは当該研究の結果の最終の公表について報告された日から3年を経過した日のいずれか遅い日までの期間とする。保管期間を経過した文書および記録は、個人情報や機密情報の漏洩がないように細心の注意を払い廃棄する。コンピュータ上およびUSBメモリなどの電磁的記録媒体にある情報は完全に削除し、紙媒体はシュレッダーにて裁断し廃棄する。

1）研究計画書

2）説明文書・同意書・同意撤回書

3）症例登録票

4）同意書（署名ありの原本）

5）症例報告書（写）

6）対応表

7）倫理審査委員会へ提出した書類

8）倫理審査委員会の結果通知書および研究機関の長の指示決定通知書

9）その他本研究に関連する文書又は記録

## 16.1. 試料・情報の二次利用について

本研究に関わる研究者が本研究で得られた情報および研究データを異なる研究目的で使用する可能性または他の研究機関に提供する可能性があるが、その場合は、新たに研究計画書を作成し、倫理審査委員会で承認を得た後に実施する。インフォームド・コンセントは、研究の内容にあわせて適切な方法で行う。

## 16.2. 試料・情報のバイオバンクとしての利用

本研究では該当しない。

# **17. 研究の資金源等，研究機関の研究に係る利益相反及び個人の収益等，研究者等の研究に係る利益相反に関する状況**

## 17.1. 資金源及び財政上の関係

本研究は，横浜市立大学医学部麻酔科学教室の基礎研究費を財源として実施する。医学的な視点から行われ、特定の企業・団体の利益や便宜をはかるものではない。また、特定の企業・団体から資金援助は受けていない。

## 17.2. 利益相反

17.2.1. 研究責任者の利益相反

研究責任者である横瀬真志には、開示すべき利益相反はない。

17.2.2.研究責任者及び研究分担者等の利益相反

研究責任者及び研究分担者の利益相反については、研究機関の利益相反ポリシー及び規程等に従い研究機関にて適切に管理を行う。利益相反の管理状況については、研究責任者に報告し、報告内容に変更が生じた場合にも報告する。

# **18.　研究対象者の費用負担・謝礼について**

本研究の実施にかかる費用のうち、個々の患者に要する医療費（診察費、入院費用、薬剤費、検査代など）については、本研究で行う治療がすべて保険診療範囲であることから自己負担分を研究対象者が支払う。本研究へ参加することで研究対象者に別途金銭的負担が増えることはない。本研究では、研究対象者に対して金銭的およびそれ以外による参加謝礼並びに負担軽減費等の支払い等はしない。

# **19. 健康被害に対する補償**

本研究に参加したことにより（起因して）研究対象者に健康被害が生じた場合、研究責任者及び研究分担者は、適切な治療及びその他必要な措置を行う。この場合の治療等は保険診療として行い、自己負担分の医療費を研究対象者が支払う。

本研究は、市販の医薬品の効能・効果、用量・用法、その他の注意事項等について添付文書に記載された範囲内で使用するため、医薬品の副作用により、死亡又は後遺障害1、2級の健康障害が生じた場合は、医薬品副作用救済制度による救済給付申請の対象となる。また、本研究で行う検査は、通常診療で行う検査と同じである。以上のことから、本研究においては臨床研究賠償責任保険への加入は行わない。

# **20. 研究機関の長への報告内容及び方法**

## 20.1. 研究の経過報告

研究の経過報告を年1回、実施状況報告書に記載し報告する。

## 20.2. 研究の終了

研究が終了したとき、研究責任者は、全ての研究責任医師にその旨および研究結果の概要を文書で報告する。各施設の研究責任医師は、研究機関の長に終了の旨および研究結果の概要を文書で報告する。

## 20.3. 研究の早期中止

研究が中止または中断された場合、理由の如何を問わず、担当医師は被験者に速やかにその旨を通知し、適切な処置ならびに被験者の安全を確認するための検査等を実施する。また実施施設の長に文書で通知する。

（１）試験治療の有効性が期待できないことや安全性に対する問題が明らかとなった場合、または研究継続の意義が無くなったと判断された場合。

（２）症例登録の著しい遅れ、研究計画書からの逸脱の頻発などの理由により、研究の完遂が困難と判断された場合。

# **21. 研究の情報公開及び結果公表**

## 21.1. 研究の登録

本研究の実施に先立ち、大学病院医療情報ネットワーク臨床試験登録システム（UMIN-CTR）に登録する。研究実施計画書の変更及び研究の進捗に応じて適宜更新する。研究を終了したときは、研究の結果を登録する。

## 21.2. 研究結果の公表

研究終了後、本研究から得られた成果を速やかに学会発表あるいは論文投稿によって公表する。公表する際は、研究対象者等及びその関係者の人権又は研究者等及びその関係者の権利利益の保護のために必要な措置を講じた上で公表する。学会発表者及び論文の筆頭著者は協議の上決定する。発表者及び筆頭著者は、発表前及び投稿前に、研究責任者の審査・承認を受ける。最終の公表を行った場合は、その旨を全ての研究機関の長に報告する。

# **22. モニタリング及び監査**

## 22.1. モニタリング

本研究は軽微な侵襲の研究であるため、モニタリングは実施しないが、3ヶ月ごとに自己点検を実施する。

## 22.2. 監査

本研究では、監査を実施しない。

22.2.1. 監査の方法

該当なし

# **23．研究成果の帰属（知的財産権）**

本研究の結果として、特許権等の知的財産権が生じた場合、その権利は公立大学法人横浜市立大学に帰属する。

# **24. 研究の実施体制**

## 24.1. 研究代表者

設定なし

## 24.2. 研究事務局

横浜市立大学附属病院　麻酔科

〒236-0004　横浜市金沢区福浦3-9

事務局代表　横瀬　真志

## 24.3. 参加施設および施設研究責任医師

　　　　横浜市立大学附属病院

　　　　〒236-0004　横浜市金沢区福浦3-9

　　　　研究責任医師　集中治療部　横瀬　真志

　　　　業務内容：研究統括

## 24.4. 統計解析責任者

横浜市立大学

〒236-0004　横浜市金沢区福浦3-9

統計解析責任医師　データサイエンス研究科　ヘルスデータサイエンス専攻　水原　敬洋

業務内容：統計解析全般

＜統計アドバイザー＞

横浜市立大学　臨床統計学　助教　三枝　祐輔

## 24.5. データマネージメント責任者

　　　　横浜市立大学附属病院

　　　　〒236-0004　横浜市金沢区福浦3-9

　　　　麻酔科　田中　宏幸

　　　　業務内容：データマネージメント全般

## 24.6. 外部委託機関

該当なし

# **25. 文献**

1. 公益社団法人日本麻酔科学会　2018年　麻酔関連偶発症例調査結果報告（日本麻酔科学会会員専用ホームページよりアクセス）
2. S.Sudfeld, S. Brechnitz, J. Y. Wagner, P. C. Reese, H. O. Pinnschmidt, D. A. Reuter and B. Saugel Post-induction hypotension and early intraoperative hypotension associated with general anaesthesia.
3. E M Wesselink, T H Kappen, H M Torn, A J C Slooter, W A van Klei Intraoperative hypotension and the risk of postoperative adverse outcomes: a systematic review Br J Anaesth. 2018; 121:706-721.
4. C C Hug Jr, C H McLeskey, M L Nahrwold, M F Roizen, T H Stanley, R A Thisted, C A Walawander, P F White, J L Apfelbaum, T H Grasela, et al. Hemodynamic effects of propofol: data from over 25,000 patients. Anesth Analg. 1993;77 (4 Suppl): S21-9.
5. Dundee JW, Robinson FP, McCollum JS, Patterson CC. Sensitivity to propofol in the elderly. Anaesthesia. 1986; 41: 482-5.
6. M Doi, K Morita, J Takeda, A Sakamoto, M Yamakage, T Suzuki. Efficacy and safety of remimazolam versus propofol for general anesthesia: a multicenter, single-blind, randomized, parallel-group, phase IIb/III trial. J Anesth. 2020; 34: 543-553.
7. M Doi, N Hirata, T Suzuki, H Morisaki, H Morimatsu, A Sakamoto. Safety and efficacy of remimazolam in induction and maintenance of general anesthesia in high‑risk surgical patients (ASA Class III): results of a multicenter, randomized, double‑blind, parallel‑group comparative trial. J Anesth. 2020; 34: 491-501.
8. 日本版敗血症診療ガイドライン2016 (The Japanese Clinical Practice Guidelines for Management of Sepsis and Septic Shock 2016) Journal of the Japanese society of Intensive Care Medicine, 24 Sup 2, 2017.
9. 日本高血圧学会高血圧治療ガイドライン作成委員会 日本高血圧診療ガイドライン2019.　ライフサイエンス出版. P52-53
10. Rockwood K, Song X, MacKnight C, et al: A global clinical measure of fitness and frailty in elderly people. CMAJ 2005; 30: 489-95
11. Memis D, Turan A, Karamanlioglu B, Sut N, Pamukcu Z. The use of magnesium sulfate to prevent pain on injection of propofol. Anesth Analg. 2002; 95: 606–8.
12. 総務省統計局ホームページ　https://www.stat.go.jp/data/topics/topi1031.html　（2020/11/02閲覧）
13. Hude Quan, et al. Updating and validating the Charlson comorbidity index and score for risk adjustment in hospital discharge abstracts using data from 6 countries Am J Epidemiol. 2011 Mar 15;173(6):676-82.

# **26. 付録**

アネレム^🄬^（レミマゾラム）添付文書

プロポフォール添付文書

西暦　　　　年　　月　　日

　　　　　　　　　　　様

**人を対象とする医学系研究の参加と協力のお願い**

研究名：高齢者に対するプロポフォールを対照としたレミマゾラムの低血圧の発生頻度を検討する並行群間ランダム化比較

１　研究の許可を受けていること

臨床研究により新しい治療法を確立することは大学病院の使命であり、患者さんのご協力により成し遂げることができるものです。今回参加をお願いする臨床研究は、実際の診療に携わる医師が医学的必要性・重要性に鑑みて、立案・計画して行うものです。

担当医師から十分に説明を受け、よく理解した上で、自由意思に基づいて研究に協力するかどうかを判断してください。参加してもよいと思われた場合には、同意文書に署名してください。なお、この研究については人を対象とする医学系研究倫理委員会の許可を得ています。

２　研究機関及び研究責任者

横浜市立大学附属病院　集中治療部　講師　横瀬　真志

３　研究の目的及び意義

全身麻酔導入薬のプロポフォールは入眠までの効果の速さや副作用・禁忌の少なさなどから本邦においては最も使用されている薬剤です。しかし、麻酔導入使用時には低血圧が生じやすい副作用があります。全身麻酔中の重度の低血圧は短時間でも臓器障害の発生や死亡率との関連性が指摘されています。本邦で世界初の認可となった全身麻酔導入薬であるレミマゾラム（アネレム®）はプロポフォールよりも低血圧発生頻度が少ない可能性が指摘されていますが、高齢患者さんで本当に低血圧が少ないかどうかは明らかではありません。本研究の目的は80歳以上の高齢患者さんでレミマゾラムとプロポフォールの使用で生じる低血圧の発生頻度を比較する事です。

４　研究の方法及び期間

　(1) 研究の方法

まず参加された場合に患者さんご自身に何か特別なことをしていただく事項はありません。医療者側が全身麻酔を始めるときに用いる薬剤をあらかじめ無作為に決めて（従来薬であるプロポフォールか新薬のレミマゾラム）、全身麻酔を始めます。どちらの麻酔薬が投与されるかについては、麻酔開始前および麻酔終了後のいずれにおいてもお知らせすることが出来ません。お休みになられるまで何度かお声をかけさせていただきます。お休み後は決まった計画書通りに麻酔薬の投与と気管挿管を行います。気管挿管3分後までは頻回に血圧測定を行い、我々が最も明らかとしたい低血圧発生の有無を記録します。それ以降は通常通り最適と思われる麻酔管理を担当麻酔科医師が行います。

翌日の回診で、有害事象の発生がないことを確認いたしますが、これに関しましても通常の診療と比較いたしまして何ら変わりません。

　(2) 研究の期間

　　本研究の研究期間は、2021年2月15日から2025年3月31日までの予定です。

５　研究対象者として選定された理由

心臓や大血管以外の手術を全身麻酔で受ける80歳以上の患者さんが対象です。さらに大きな合併症をお持ちでなく手術リスクが比較的低い患者さんを対象としています。

６　負担並びに予測されるリスク及び利益

　　本研究で用いる薬剤はいずれも全身麻酔の導入薬として保険適用されています。研究に参加いただくための直接的な経済上の負担はありませんが、お薬の値段が異なるためお支払いいただく医療費に差が生じます（具体的には1割負担の方で150-180円程度の違い）。患者さんはどちらのお薬を使用するかは事前には知ることが出来ないため、より安価な医療を選択できない不利益があります。新薬の使用方法は添付文書に沿っており、麻酔薬としての効果はプロポフォールと同等ですので研究参加によるリスクの増加（麻酔がかからない等）はありません。むしろ新薬のレミマゾラムは低血圧発生が少ないことが予想されますので、より安全な麻酔を受けられる可能性があります。また、プロポフォールが投与される場合でも血圧測定は通常より頻回測定となり、より早く低血圧に対する治療を受けられる可能性があります。

７　撤回できること

この研究に協力していただくことに同意された後、または参加途中であっても、いつでも辞退することができます。研究への参加をやめたいと思われた場合は、その理由にかかわらず、「同意撤回書」（別紙）を用いて、参加を中止することができます。ただし、同意の撤回をお申し出いただいた時点で、すでに研究結果が公表されていたときなど、データから除けない場合があります。

８　不利益を受けないこと

この研究への参加をご辞退されても不利益になるようなことは一切なく、本来の治療方針に沿った治療を受けることができます。

９　他の治療方法について

　　通常の全身麻酔導入に用いる薬剤を担当麻酔科医師が適宜判断して使用します。通常はプロポフォールを用いた麻酔導入で、血圧測定の回数は研究参加時ほど頻回には測定されないことが予想されます。

10　資料の閲覧

すべての研究対象者等は、さらに詳しい研究計画書及び研究の方法に関する資料を入手又は閲覧することができます。その場合、他の研究対象者等の個人情報等は保護し、また研究の独創性の確保に支障がない範囲で行います。入手又は閲覧の方法は、下記に記載されている研究責任者への連絡を行っていただき、研究責任者および分担者の複数名で協議の上で閲覧可能な資料の提供を行います。

11　個人情報等の取り扱い

ご参加いただいた場合、あなたの診療記録の一部を、本研究の目的のために使用いたします。具体的には、氏名、生年月日、カルテID等あなたを特定できるような個人情報を削除し、年齢、性別、診察の結果得られる診療情報を使用させていただきます。

研究に使用する際には、研究用の番号（識別番号）を付けて取り扱います。あなたと識別番号を結びつけるものとして対応表と呼ばれるものを作成します。その対応表及びその他の情報等を電子媒体で保管する場合は、パスワードを設定した電子ファイルで、横浜市立大学附属病院　麻酔科のインターネットに接続できないパソコンで保存します。このパソコンが保管されている部屋は、入室が管理されており第三者が立ち入ることはできません。本研究によって得られた情報も対応表と同様に管理します。

12　試料・情報の保管及び廃棄の方法

情報は、少なくとも本研究の終了日から5年後又は本研究の結果の最終の公表について報告した日から３年後のいずれかの遅い日までの期間、上記11項の記載の通り厳重に管理します。電子データはパスワードを設定した上で、インターネットから独立したパソコンまたはUSBメモリ等の電磁的記録媒体にて保管し、使用していない時は施錠可能な保管庫で厳重に保管管理します。紙媒体のものも同様に施錠可能な保管庫で管理します。また情報は、研究終了後コンピュータ上およびUSBメモリなどの電磁的記録媒体にある情報は完全に削除し、紙媒体はシュレッダーにて裁断するという方法ですみやかに廃棄処分いたします。

13　利益相反

　　利益相反とは、外部との経済的な利益関係等によって、研究データの改ざん、特定企業の優遇など研究が公正かつ適切に行われていないと第三者から懸念されかねない事態のことを指します。本研究は、横浜市立大学医学部麻酔科学教室の基礎研究費を財源として実施されるため、特定の企業からの資金提供を受けておらず、特定の企業の利益を優先させて、あなたの治療方針を変えてしまったり、研究の公正さを損なったりすることはありません。なお、本研究における当院の研究者の利益相反については、本学の臨床研究利益相反委員会で審査され、適切に管理されています。

14　研究対象者等及びその関係者からの相談

この研究について知りたい事や、心配な事がありましたら、遠慮なく担当医師にご相談下さい。ただし、他の研究者等の個人情報や研究者の知的財産権の保護等の観点から、回答ができないことがあります。その場合は担当医師から説明をいたします。

15　費用について

　　通常の診療でかかる費用（保険診療の一部負担金）はこの研究へご協力いただかない場合と同様にご負担いただきます。またこの研究への参加謝礼はありません。

16　研究実施後の医療提供

　　この研究に伴う介入は全身麻酔中のみですので、術後の治療方法などに影響を及ぼすことはありません。研究に参加されない場合と同様の医療が提供されます。

17　偶発的所見の取り扱い

偶然にこの研究とは関係のない、重大な病気（麻酔中のバイタルサインや薬剤に対するアレルギーなど）との関連が見つかった場合には、麻酔科医師もしくは診療科医師からお知らせいたします。

18　健康被害への補償

本研究は、これまでの報告に基づいて科学的に計画され、慎重に行われます。

もし研究の期間中あるいは終了後に本研究に起因してあなたに副作用などの健康被害が生じた場合には、医師が適切な診察と治療を行います。

19　将来の研究の可能性

今回の研究で得られた診療情報を用いた二次的な解析を行う可能性があります。その際には新たな診療情報の収集は行われず、患者さんへの新たな身体的・経済的負担の発生はありません。また、当院倫理委員会に研究計画書を提出し、倫理的な問題がないことを確認の上で行うこととなります。

20　関係者が試料・情報を閲覧すること

研究の適正な実施のために、倫理審査委員会が、必要な範囲内において当該研究対象者に関する試料・情報を閲覧することがあります。

21　研究成果の公表について

　　研究責任者は介入を行う研究について、国立大学附属病院長会議他が設置している公開データベースに、研究の概要をその実施に先立って登録し、研究計画書の変更及び研究の進捗に応じて適宜更新します。また研究を終了したときは、遅滞なく研究の結果を登録します。ただし、人権や権利利益の保護のため非公開となる場合もあります。

　　研究成果については、今後の治療に役立てるため、学術集会や論文などで発表・公表させていただくことがございます。しかし、氏名等の個人情報は、一切公表されることはなく堅く守られることを保証します。

22　知的財産権について

この研究の成果により、特許権等の知的財産権が生じる場合がありますが、その権利は全て、研究機関側に帰属し、あなたには生じないことをご了承ください。

　説明を行った医師等　　　　　　　科　　　　　　　　　　　　　　㊞

連絡お問い合わせ先：

横浜市立大学附属病院　集中治療部

研究責任者　　　　横瀬　真志

電話番号　　　　　045-787-2800（代）

なお、上記連絡先と連絡が取れないときは、横浜市立大学附属病院　臨床研究推進課倫理担当　　電話045-370-7627まで、お問い合わせ下さい。

No.

同　意　書

私は「研究名：全身麻酔を受ける高齢者に対するレミマゾラムの低血圧発生頻度：二重盲検ランダム化比較試験」（研究責任者：集中治療部　横瀬　真志）への参加協力について、説明者　　　　　科　　　　　　　　　により別紙「試験研究の参加と協力のお願い」に基づき以下の説明を受けました。

□１　研究の許可を受けていること □12　試料・情報の保管及び廃棄の方法

□２　研究機関及び研究責任者 □13　利益相反（起こり得る利害の衝突）

□３　研究の目的及び意義 □14　研究対象者等及びその関係者からの相談

□４　研究の方法及び期間 □15　費用について

□５　研究対象者として選定された理由 □16　研究実施後の医療提供

□６　負担並びに予測されるリスク及び利益 □17　偶発的所見の取り扱い

□７　撤回できること □18　健康被害への補償

□８　不利益を受けないこと □19　将来の研究の可能性

□９　他の治療方法について □20　関係者が試料・情報を閲覧すること

□10　資料の閲覧 □21　研究成果の公表について

□11　個人情報等の取り扱い □22　知的財産権について

上記の説明を受け、十分に理解しましたので、自由意思により本研究に参加協力いたします。

　研究成果については個人のプライバシーが守られることを条件に学会・論文等で発表・公表されることに同意します。

　また、研究の適正な実施のために、倫理審査委員会が診療に関する記録を閲覧することに同意します。

参加協力の内容：

横浜市立大学附属病院 病院長

西暦　　　年　　月　　日

協力者氏名

参加協力を同意された研究に関する疑問・不安な点などは下記にお問い合わせ下さい。

連絡お問い合わせ先：

横浜市立大学附属病院　集中治療部

研究責任者　　　　横瀬　真志

電話番号　　　　　045-787-2800（代）

なお、上記連絡先と連絡が取れないときは、横浜市立大学附属病院　臨床研究推進課倫理担当

電話045-370-7627まで、お問い合わせ下さい。
