# Supplementary material for: Hypotension after general anesthesia induction using remimazolam in geriatric patients: Protocol for a double-blind randomized controlled trial
Source: PLoS One. 2022 Sep 30;17(9):e0275451. doi: 10.1371/journal.pone.0275451 (PMC9524631; doi:10.1371/journal.pone.0275451)
Supplement: Attachment — Submitted filename: Plos one-for authors.docx [file pone.0275451.s005.docx]

Dear colleagues

Thank you for submitting the manuscript entitled: Hypotension after general anesthesia induction using remimazolam in geriatric patients: Protocol for a double-blind randomized controlled trial to PLOS ONE journal. I read this amazing manuscript and I think you should consider the following points about your manuscript.

1. In the method you wrote, patients aged more than 80 years therefore is it possible these patients were pregnant? As you wrote in the exclusion criteria?
2. How could you blind this study because the colors of propofol and remimazolam are different, please explain more about this.

Best regards.
