# Supplementary material for: Hypotension after general anesthesia induction using remimazolam in geriatric patients: Protocol for a double-blind randomized controlled trial
Source: PLoS One. 2022 Sep 30;17(9):e0275451. doi: 10.1371/journal.pone.0275451 (PMC9524631; doi:10.1371/journal.pone.0275451)
Supplement: Attachment — Submitted filename: Dear respectable editor.docx [file pone.0275451.s006.docx]

Dear respectable editor

I read the manuscript that was well defined and good writing also, a novel subject in geriatric anesthesia. However, it needs a minor revision.

Best regards
