# Supplementary material for: Hypotension after general anesthesia induction using remimazolam in geriatric patients: Protocol for a double-blind randomized controlled trial
Source: PLoS One. 2022 Sep 30;17(9):e0275451. doi: 10.1371/journal.pone.0275451 (PMC9524631; doi:10.1371/journal.pone.0275451)
Supplement: Attachment — Submitted filename: Response_to_reviewer.docx [file pone.0275451.s007.docx]

Dear Editor,

We greatly appreciate the editor and reviewers’ comments regarding our manuscript, titled **"Hypotension after general anesthesia induction using remimazolam in geriatric patients: Protocol for a double-blind randomized controlled trial**” (PONE-D-22-13552). We have revised our manuscript according to the comments by the reviewers. Our responses to the comments are described below in a point-by-point manner.

Reviewer #1:

Reviewer’s comment:

1. In the method you wrote, patients aged more than 80 years therefore is it possible these patients were pregnant? As you wrote in the exclusion criteria?

Our response:

We agree with the reviewer’s comment that the exclusion criterion was not correctly phrased. We have removed the term “pregnancy” from the exclusion criterion, and renumbered the list as follows (Lines 109-119 of the revised manuscript).

“The exclusion criteria are: (1) cerebral aneurysm or thoracic/abdominal aortic aneurysm; (2) predicted/history of difficulty in airway management; (3) allergies to drugs used in the study, i.e., remimazolam, propofol, and rocuronium; (4) undergoing maintenance dialysis; (5) severe liver dysfunction, i.e., Child–Pugh class C; (6) untreated/unstable ischemic heart disease; (7) severe aortic/mitral valvular disease; (8) arterial fibrillation, multiple atrial premature contraction, or ventricular premature contraction; (9) congestive heart failure (New York Heart Association classification Ⅲ or Ⅳ); (10) body mass index ≥30 kg.m^-2^; (11) regular use of antipsychotics or antidepressant drugs; (12) mean blood pressure (MBP) <70 mmHg before general anesthesia induction; (13) failed first attempt of tracheal intubation; and (14) judged inappropriate for participation by the researchers.”

Reviewer’s comment:

2. How could you blind this study because the colors of propofol and remimazolam are different, please explain more about this.

We thank the reviewer for this constructive comment that will help us clarify our research protocol further. We have added a detailed description of the blinding process as follows (Lines 249-258 of the revised manuscript).

“A syringe and a connected extension tube, filled with trial drugs and mounted on a syringe pump, will be prepared by unblinded researchers. All of these components will be covered by an opaque plastic bag outside the operating room and brought into the operating room by an unblinded researcher. After laying the patient on the operating table, the entire IV line and puncture site on the patient's forearm will be covered with towels and opaque plastic sheets. The extension tube will be connected to a triple three-way stopcock in the IV line under the towels. The syringe pump will be operated from inside the opaque plastic bag. Subsequently, blinded researchers and attending anesthetists will enter the operating room and general anesthesia induction will be initiated.”
